# Harmonized Life-Cycle Inventories of Nanocellulose and Its Application in Composites

**DOI:** 10.1021/acs.est.3c04814

**Published:** 2023-11-15

**Authors:** Seth Kane, Sabbie A. Miller, Kimberly E. Kurtis, Jeffrey P. Youngblood, Eric N. Landis, W. Jason Weiss

**Affiliations:** † Department of Civil and Environmental Engineering, University of California Davis, Davis, California 95616, United States; ‡ School of Civil and Environmental Engineering, Georgia Institute of Technology, Atlanta, Georgia 30332, United States; § School of Materials Engineering, Purdue University, West Lafayette, Indiana 47907, United States; ∥ Department of Civil and Environmental Engineering, University of Maine, Orono, Maine 04469, United States; ⊥ School of Civil and Construction Engineering, Oregon State University, Corvallis, Oregon 97331, United States

**Keywords:** cellulose nanocrystal (CNC), cellulose nanofiber (CNF), cement, plastic, life cycle assessment (LCA), nanocomposite

## Abstract

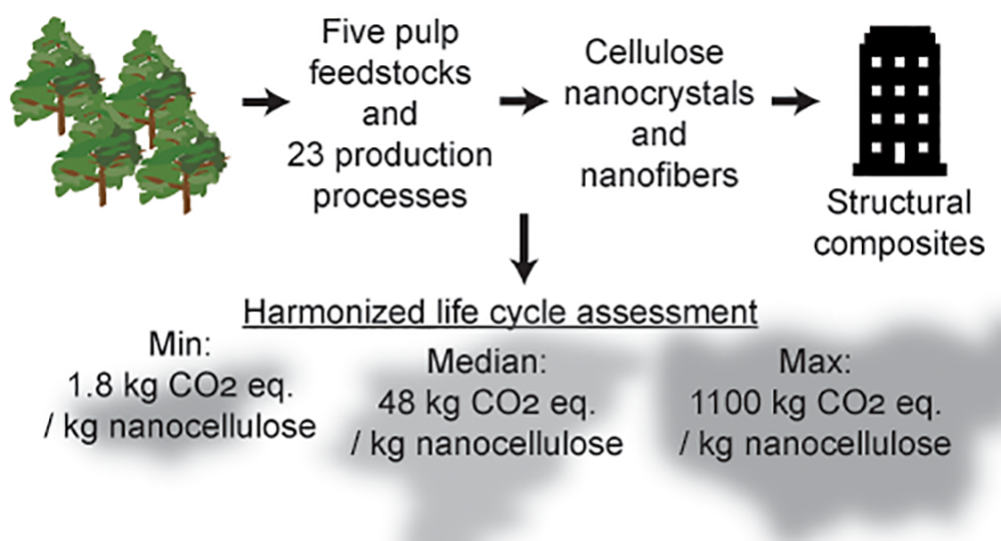

Cellulose nanocrystals (CNC) and nanofibers (CNF) have been broadly studied as renewable nanomaterials for various applications, including additives in cement and plastics composites. Herein, life cycle inventories for 18 previously examined processes are harmonized, and the impacts of CNC and CNF production are compared with a particular focus on GHG emissions. Findings show wide variations in GHG emissions between process designs, from 1.8–1100 kg CO_2_-eq/kg nanocellulose. Mechanical and enzymatic processes are identified as the lowest GHG emission methods to produce CNCs and CNFs. For most processes, energy consumption and chemical use are the primary sources of emissions. However, on a mass basis, for all examined production methods and impact categories (except CO emissions), CNC and CNF production emissions are higher than Portland cement and, in most cases, are higher than polylactic acid. This work highlights the need to carefully consider process design to prevent potential high emissions from CNCs and CNF production despite their renewable feedstock, and results show the magnitude of conventional material that must be offset through improved performance for these materials to be environmentally favorable.

## Abstract 2

Producing nanocellulose results in a broad range of greenhouse gas emissions depending on processing method, with mechanical and enzymatic processes resulting in the lowest emissions.

## 1 Introduction

Nanomaterials have facilitated profound alterations in material performance, which can alter how novel materials are engineered and used; however, increased use of nanomaterials has also driven concern about their environmental impacts as their production can lead to harmful burdens.^1−3^ One proposed pathway to reducing such environmental impacts from nanomaterials production is utilizing biogenic resources as feedstock, such as cellulose nanocrystals (CNCs) and cellulose nanofibers (CNFs). CNCs and CNFs have been broadly examined as nanomaterial additives in cementitious and polymer composites (i.e., nanocomposites).^4−7^ Findings from such experimental investigation have shown the unique microstructure and surface chemistry of these cellulosic nanomaterials result in substantial benefits to the performance of these nanocomposites relative to conventional materials for a wide range of applications.

Nanocellulose, including CNCs and CNFs, can be produced from many lignocellulosic biomass precursors and through various processes. Of the processes used, acid hydrolysis is the most commonly applied commercially and in research. In the acid hydrolysis process, typically sulfuric acid is used to hydrolyze and then esterify cellulose.^1^ This method has been widely applied to produce CNCs, resulting in high yields and predictable cellulose properties.^1^ Acid hydrolysis can also be applied with other acids, such as hydrochloric acid, to vary CNC properties. However, this is not the only method for nanocellulose production. Highly crystalline nanocellulose can be isolated with acid-free processes by oxidation. In oxidative processes, oxidation can be performed with H_2_O_2_ and UV or heat or catalyzed with 2,2,6,6-tetramethylpiperidine-1-oxyl (TEMPO) (at lab scale) or iron (commercially), resulting in cellulose with carboxyl functional groups. This process typically results in lower yields than acid hydrolysis processes, but it is often touted to be lower cost and more environmentally sustainable.^1,8^ In addition to these two primary methods, ionic, enzymatic, homogenization, and mechanical routes have been used to isolate CNCs and CNFs. Among these, mechanical processes are prevalent in commercial applications. However, these routes typically result in larger-sized or less crystalline nanocellulose,^1^ and are typically applied to produce CNFs rather than CNCs.^7^ Oxidative processes can also be applied as pretreatments for other processes, for example, TEMPO-oxidation pretreatment for mechanical processes.^9^

CNCs and CNFs have been evaluated as nanomaterial additives to cementitious and polymeric composites. While CNCs and CNFs have high modulus (110–220 GPa) and tensile strength (7500–7700 MPa), bonding and dispersion also play an important role in their performance in composites.^10^ Due to these mechanical characteristics, their high surface area, and surface chemistry, nanocellulose can contribute to increasing mechanical performance in cementitious and polymeric composites. In cementitious composites, adding low quantities (typically 0–4 wt %) of CNCs or CNFs results in increased compressive and flexural strength, improved rheology, increased hydration rates (due to nucleation effects leading to improved early reaction kinetics) and overall hydration degrees.^11−15^ For example, Fu et al. found that adding 0.5 wt % CNC to Portland cement resulted in a 19% increase in compressive strength.^10^ In polymer composites, CNCs and CNFs are typically added at up to 10 wt %, typically resulting in increased crystallinity, mechanical strength, and stiffness. For example, a 21% increase in tensile strength and a 24% increase in tensile modulus of polylactic acid (PLA) with 5 wt % CNF addition reported by Jonoobi et al.,^16^ attributed to the high aspect ratio of CNCs, high strength of CNCs, and strong interfacial interactions between CNCs and polymers.^7^ Whereas improved performance in cement is primarily due to changes in reaction kinetics and increased cement hydration, improvements in polymers are attributed to nucleation of crystallization and stronger bonding between polymer and nanocellulose. Notably, because of their biogenic origin, CNCs and CNFs have been widely examined in biodegradable polymers, such as PLA, as they may biodegrade at the material end-of-life, unlike common mineral- or petroleum-derived nanofillers.^7^ At higher loadings of nanocellulose, performance in both cementitious and polymeric composites is limited by the agglomeration of nanocellulose fibers. Surface functionalization of nanocellulose with groups such as silane or carboxyl can further improve the interactions of nanocellulose with cement and polymers, leading to improved bonding and dispersion.^17−19^

As noted, many past studies of CNCs and CNFs have proposed these materials to be “sustainable” or “green” due to their renewable biomass precursor.^4,5^ However, using biogenic resources does not unilaterally result in low environmental impact materials. Instead, systematic methods, such as life cycle assessments (LCAs), must be performed to quantitatively determine the impacts associated with products. In consideration of CNCs and CNFs, several LCA studies have examined the environmental impacts of producing these materials with several processes, including acid hydrolysis, homogenization, TEMPO-oxidation, deep eutectic solvent (DES), etherification, mechanical, carboxymethylation, and enzymatic processes.^9,20−26^ Looking at results across these studies show high variation in emissions both within individual processes (e.g., 31–122 kg CO_2_ eq/kg nanocellulose for acid hydrolysis processes) and between processes, with a total variation in greenhouse gas (GHG) emissions of 0.79–1160 kg CO_2_ eq/kg nanocellulose. However, the scopes of assessment and modeling assumptions for these studies also varied greatly (e.g., which biomass resource was used, what energy grid was used, and what aspects of production were considered inside and outside the scope of assessment). This high variation in modeling between studies is a critical gap in understanding the GHG emissions attributed to the production of CNCs and CNFs. Differences in inventories, system boundaries, and assumptions between studies make it challenging to compare the results of these individual studies directly. Such differences remain a critical gap to determining the best pathways to produce low environmental impact CNCs and CNFs and limit the ability to determine which biogenic resources and processes should be prioritized to mitigate environmental impacts.

In this work, we harmonize life cycle inventories for producing CNCs and CNFs to investigate the GHG and key air pollutant emissions associated with their production considering various processing methods and biogenic precursors. We then use findings to discuss pathways for limiting environmental impacts from their production and to understand environmental impact benefits and drawbacks, including the role of mechanical strength improvements, for their use as additives in cementitious and polymer composites where CNCs and CNFs have been extensively studied as additives.

## 2 Methods

### 2.1 Goal and Scope

**Figure 1:**
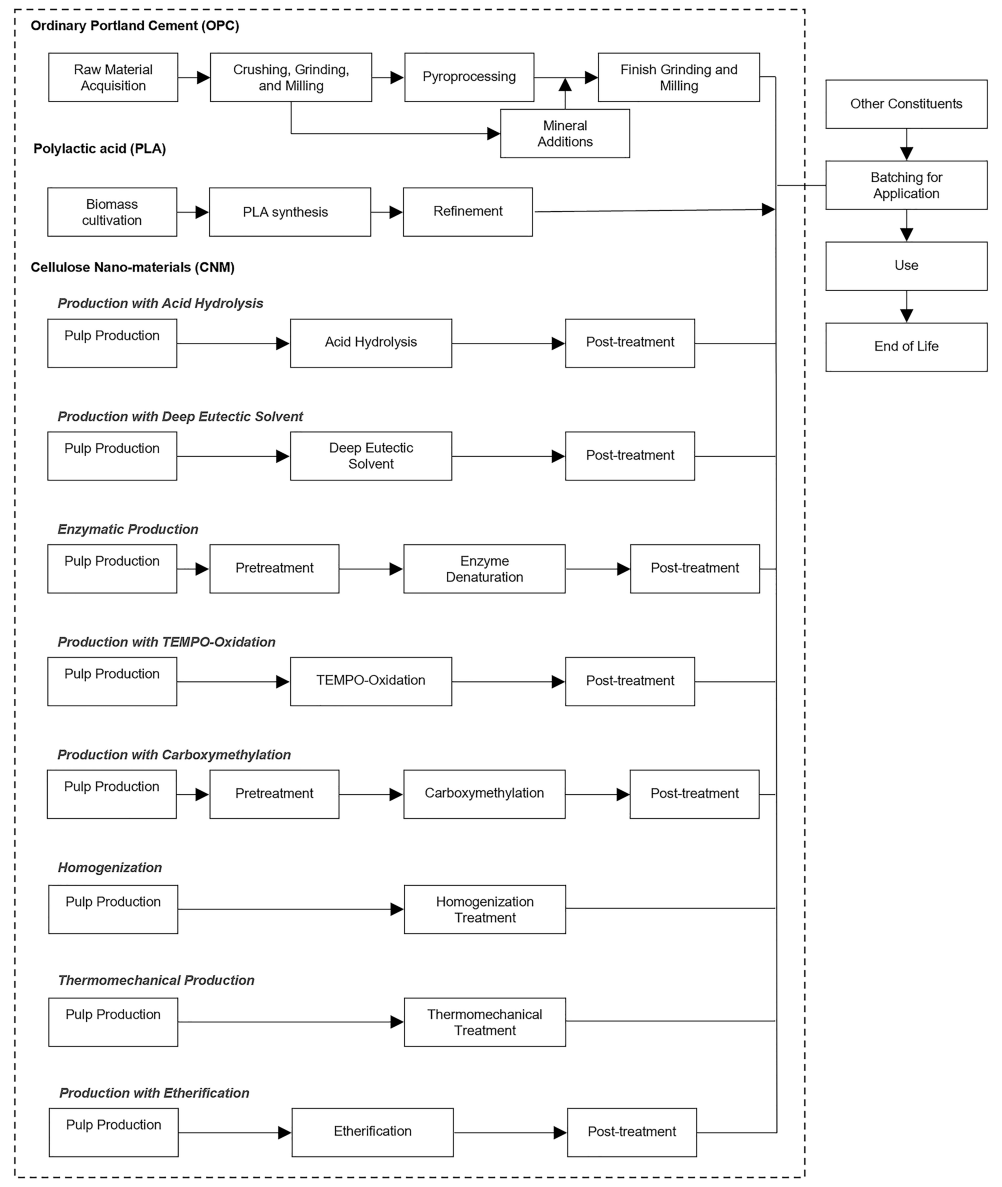
Simplified process flow diagrams of processes considered within the scope of this assessment (within the dashed lines). A complete list of process steps for each process is given in SI Table S1.

Here, we examine the cradle-to-gate production of CNCs and CNFs (see Figure 1). This scope of assessment includes raw material acquisition, transportation of goods, and processing of resources to produce the nanomaterials. The use phase and end-of-life phases are outside the scope of analysis. Comparisons of processes are made with a functional unit of 1 kg. For composite materials of nanocellulose with cementitious or PLA composites, comparisons are additionally made on a strength basis (i.e., impact per kg per unit strength ((kg CO_2_-eq/kg composite)/MPa) to highlight the role that changes in performance may play in environmental impacts. Comparisons are made for compressive strength for cementitious composites and tensile strength for PLA composites.

Five biogenic resources and eight processing methods are considered (see Section 2.2). This work’s Comparisons are based on the Intergovernmental Panel on Climate Change 2013 global warming potentials (5^th^ Assessment Report, AR5).^27^ Additionally, we draw comparisons based on cumulative energy demand (CED) and flows, including carbon monoxide (CO), volatile organic compounds (VOC), sulfur dioxide (SO_*x*_), nitrogen oxides (NO_*x*_), particulate matter <2.5 μm (PM_2.5_), and lead (Pb) tabulated by the SimaPro software.^28^

To perform this assessment, we leverage existing inventories from the literature (Table 1). A critical contribution of this effort is to harmonize modeling assumptions so comparisons can be better drawn across these alternatives. We use these inventories from the literature to determine quantities of flows. However, to harmonize the inventories, assumptions regarding energy sources, modes of transportation and distance, resource acquisition, and other inputs are held constant.

**Table 1:** Summary of Inventory Sources and Notes Used in Original Studies and in the Harmonized Inventories

cellulose	reference	key process	pulp source	allocation	feedstock transportation distance (km)	waste flows	scale	inventory notes
CNC	Gu et al.^20^	acid hydrolysis	bleached kraft pulp	waste outflows were considered to reduce net impacts by offsetting primary production	1400	outflows assumed to offset primary production of other products	pilot	
CNC	Teh et al.^23^	acid hydrolysis; TEMPO-oxidation	empty fruit bunch	no allocation reported	not reported	not reported	laboratory	We assumed all nonspecified energy inputs are electricity; inventories are missing cellulose source and waste flows, a 1:1 input of cellulose source to nanocellulose output is assumed.
CNC	de Figueirêdo et al. ^22^	acid hydrolysis	cotton pulp, coconut fibers^a^	all emissions allocated to nanocellulose	excluded	effluent treatment excluded	laboratory	We assumed all energy not noted as thermal energy is electricity; We assumed all water noted as deionized water to remain consistent with other inventories.
CNC	Zargar et al.^24^	acid hydrolysis; deep eutectic solvent (DES) pretreatment	thermomechanical pulp	all emissions allocated to nanocellulose	2684	wastewater, waste chemicals, and residual biomass included	laboratory	Some inputs called for inventories that were unavailable. We assumed the same proxy inventories where applicable, and used inventories for similar compounds otherwise.
CNF	Arvidsson et al.^25^	enzymatic pretreatment; carboxymethylation; homogenization	elementary chlorine free, totally chlorine free, and unbleached, kraft pulp, chlorine bleached sulphite pulp	all emissions allocated to nanocellulose	1574–4400, multiple transportation modes considered.	waste not reported	pilot	missing mass inputs for nonconverted pulp (i.e., a 1:1 ratio of pulp in to CNF out is unlikely).
CNF	Stampino et al.^9^	enzymatic pretreatment; TEMPO-oxidation	cotton linters, bleached mixed hardwood kraft pulp, industrial waste sludge	all emissions allocated to nanocellulose	200	missing solid waste	laboratory	
CNF	Li et al.^26^	etherification; TEMPO-oxidation	kraft pulp	no allocation reported	excluded	missing solid waste; liquid waste combusted for energy	laboratory	
CNF	Sun et al.^21^	mechanical (wet disc milling)	wood pulp	no allocation reported	50–150	missing waste	pilot	missing mass inputs for nonconverted pulp (i.e., a 1:1 ratio of pulp in to CNF out is unlikely).
**CNC and CNF**	**harmonized inventory**	**all**	**bleached kraft, kraft, and thermos-mechanical pulp cotton linters, industrial waste**	**all emissions allocated to nanocellulose**	**200**	**wastewater, waste chemicals, and waste biomass all excluded**		

a Note: The inventory for coconut fibers was not utilized as it was more dissimilar from the pulp sources considered herein and did not lend itself to the homogenization efforts being performed.

### 2.2 Inventories

Using the types and quantities of flows for inventories for CNCs and CNFs from the existing literature (Table 1), we harmonize inventories from multiple sources by utilizing coherent inputs. These include:

- We use consistent inventories for raw material resources and the same mixes for energy and thermal resources. We accomplish this by using recent inventory models (from 2016, ecoinvent database^29^); this single database allowed us to capture relevant resource, process, and energy demands while mitigating variability between life cycle databases.
- We implement consistent transportation assumptions throughout CNC and CNF production methods. We accomplish this through two mechanisms: (1) the distance the pulp is transported is set as 200 km; and (2) we use the “market for” inputs (based on the ecoinvent database^29^) for resources, which captures typical transportation distances associated with these commodities to their market user.
- All inventories were compared across five commonly examined pulp sources: bleached kraft pulp, cotton linters, industrial waste, kraft pulp, and thermo-mechanical pulp. Kraft pulp was discussed in detail as the most commonly examined pulp source. The literature suggests pulp sources can notably lead to variations in environmental impacts.^9^ Therefore, we include the same permutation of inventories with each alternative cellulose source to address how the impacts from these CNC and CNF processes may vary if a less commonly examined pulp source were used. We note the homogenization of pulp sources for each inventory involves changing pulp sources from that of the original studies, which can add some uncertainty and may require changes in process design that were not modeled.
- We homogenized assumptions for the allocation of secondary resources and avoided products. Namely, in this work, we do not consider reduced impacts for CNCs or CNFs if a byproduct is generated during their production, even where that could offset another product’s conventional production. Further, we do not consider impacts from primary processes if a residue of those processes is used as an input for CNC or CNF production.
- As some harmonized inventories do not report waste flows, treatment of waste flows was omitted for all inventories to allow for standardized comparisons. However, when available, these flows are reported in the tabulated inventories in Supporting Information (SI) Tables S2–S24.

As noted, here we use unit processes from the ecoinvent database.^29^ Global (GLO) average inputs were used where possible; however, in cases where GLO data were unavailable, rest of world (RoW) inputs were used.

We note that while most studies examined utilize nanocellulose production data from the laboratory scale, three studies utilize pilot scale data. Due to limitations in data availability, we do not attempt to harmonize differences between laboratory and pilot scale.

From the eight sources listed in Table 1, we extracted inventories for 18 processes and five feedstocks (see SI Table S1). For three processes—DES,^24^ mechanical,^21^ and homogenization^25^—multiple sets of process parameters were considered, resulting in 23 process designs and 115 total combinations of process, process parameters, and feedstock. A complete list of process parameters and inventories is shown in the (SI Tables S1–S24). Simplified process flow diagrams for the process types of cellulose nanomaterial production considered are presented in Figure 1. Due to interest in the ability of nanocellulose to act as an additive that can limit GHG emissions from concrete and plastic production, here we compare the CNC and CNF models to the RoW Portland cement model and the RoW polylactic acid (PLA) model from the ecoinvent database^29^ and again using “market for” data to maintain consistency. Additions of CNC or CNF to these materials are discussed over replacement levels commonly examined in the literature, 0–4 wt % for cement and 0–20 wt % for PLA.^10,30,31^

As part of this work, trends are also examined through statistical analyses conducted in Minitab (v. 21.2), and the critical alpha was set a priori to 0.05 for all analyses. A Kruskal–Wallis test was performed to examine differences in GHG emissions by nanocellulose type (CNC or CNF), as the data did not meet the assumptions of normality or heteroskedasticity for an ANOVA. A two-factor ANOVA was performed to examine the impact of feedstock and critical process type on CNC and CNF GHG emissions. We note data does not meet assumptions of normality and heteroskedasticity, and sample sizes are unequal between process type groups, limiting the statistical power of this test. Post hoc testing was performed with the Bonferroni Method to examine differences between individual processes.

## 3 Results and Discussion

**Figure 2:**
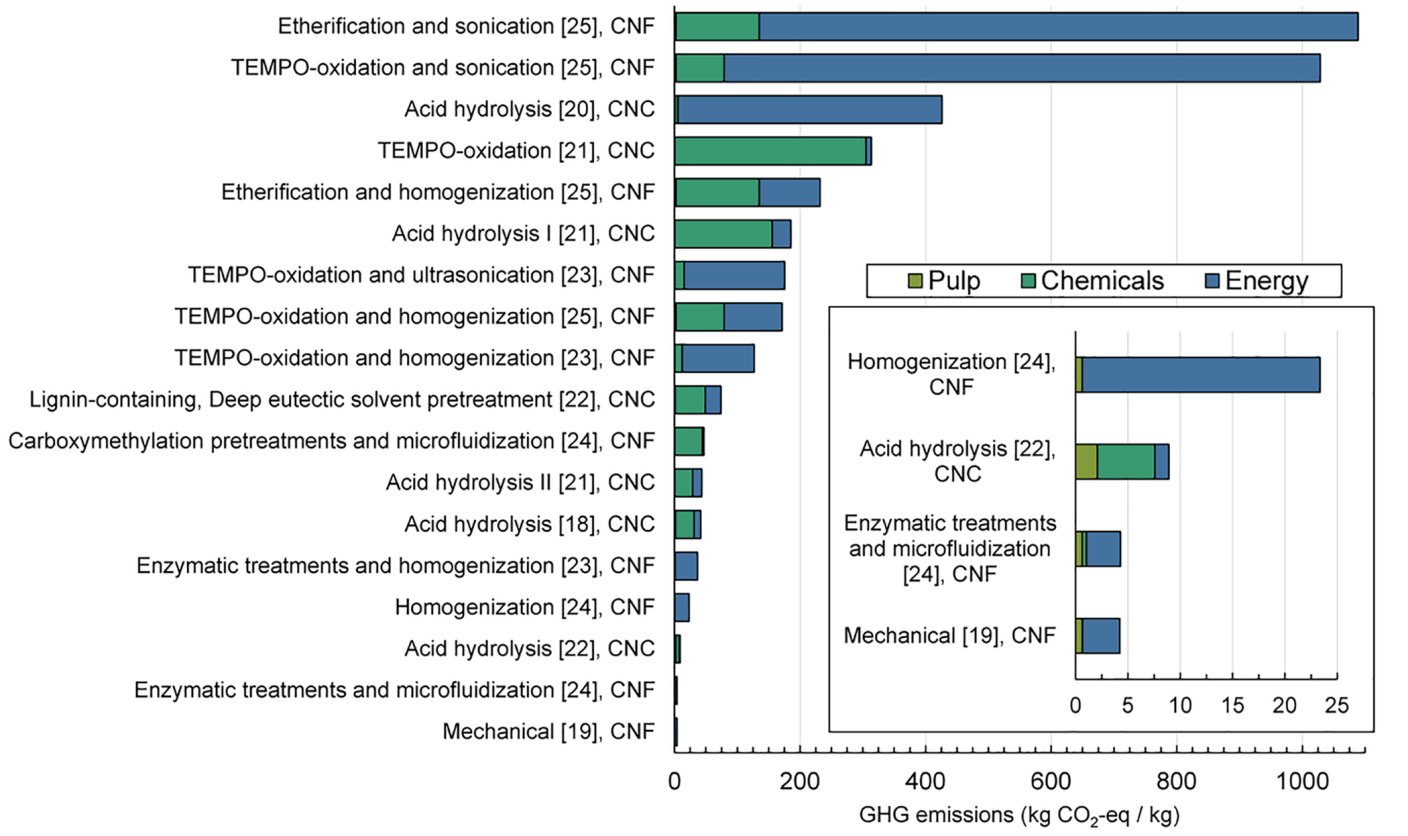
Distribution of greenhouse gas emissions for 115 combinations of CNC and CNF feedstock and process modeled. Here (●) represents the median, the upper and lower bounds of the boxes represent the 25^th^ and 75^th^ percentiles, and the upper and lower bounds of the whiskers represent the 5^th^ and 95^th^ percentiles. (a) Comparison of CNC and CNF GHG emissions. (b) Comparison of primary CNC or CNF production process. A, B, and C labels in (b) mark processes with significantly different GHG emissions. Processes that do not share a letter have significantly different GHG emissions. Note sample sizes differ between processes (SI Table S25).

Even with the harmonization of inventories, there are wide variations in the GHG emissions of CNCs and CNFs depending on process and inputs (Figure 2a). A Kurskal-Wallis test shows moderate evidence for a difference in GHG emissions between CNC and CNF production (*p* = 0.06). CNFs show a wide dispersion in GHG emissions from production (between 1.8–1100 kg CO_2_-eq/kg CNF). While the dispersion of GHG emissions from CNC production is narrower than that of CNFs, there is still notable variation (ranging between 6.8 to 430 kg CO_2_-eq/kg CNC). For the inventories modeled, CNF has a slightly lower median value of 42 kg CO_2_-eq/kg relative to 75 kg CO_2_-eq/kg for CNC. The broader distribution of GHG emissions for CNF relative to CNC is attributed to both its larger sample size (*n* = 70 for CNF and *n* = 45 for CNC) as well as the wider range of process types examined for CNF production (6 processes) than for CNC production (3 processes).

Even when considering the production of nanocellulose with similar processes, there are still notable variations in GHG emissions observed between process types (Figure 2b). For example, GHG emissions from acid hydrolysis processes vary from 6.8–430 kg CO_2_-eq/kg nanocellulose. A two-factor ANOVA considering the primary process and feedstock used found strong evidence of a difference in GHG emissions by the primary process (*p* < 0.001). Post hoc testing identified all processes with median GHG emissions <100 kg CO_2_-eq/kg nanocellulose (acid hydrolysis, DES pretreatment, carboxymethylation, enzymatic pretreatment, homogenization, and mechanical) as not significantly different. Similarly, high median GHG emission etherification and TEMPO-oxidation processes were not significantly different. Primary processes with both lower median emissions and lower ranges of GHG emissions, such as homogenization and mechanical separation, have fewer inventories (one study for both homogenization and mechanical separation) than the higher median and range of GHG emission processes, such as acid hydrolysis, or TEMPO-oxidation (four and three studies, respectively). This difference highlights the critical need for further study of these low-emission processes to determine if lower impacts are tied to the limited data or if there is similar variability as in more commonly studied processes. Post-treatment processes can have a meaningful impact on overall process GHG emissions. For example, sonication and ultrasonication post-treatment processes examined by Stampino et al. and Li et al.^9,26^ resulted in an average increase in GHG emissions of 450 kg CO_2_-eq/kg nanocellulose over homogenization post-treatment. Therefore, selecting low-energy and low-GHG emission postprocessing methods is critical in designing low-GHG emission nanocellulose production processes.

**Figure 3:**
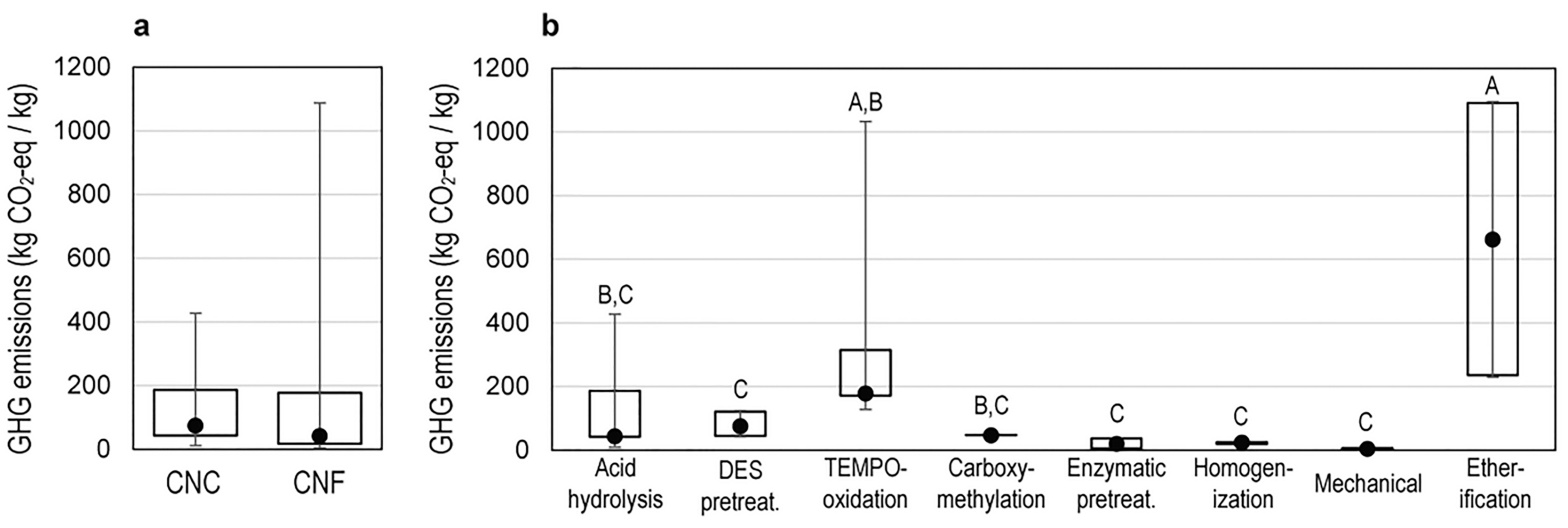
Summary of inventory sources and greenhouse gas emissions associated with producing CNCs and CNFs using kraft pulp as the cellulose source. For processes with multiple parameters examined, median values are shown. Inset shows emissions for inventories <25 kg CO_2_-eq/kg in detail. Emissions for feedstocks other than kraft pulp are shown in SI Table S25.

The lowest GHG emission processes when CNC and CNF production when modeled with kraft pulp feedstock (Figure 3) resulted from mechanical and enzymatic with microfluidization processes, leading to 4.2 and 4.3 kg CO_2_-eq/kg CNF, respectively. The highest GHG emission processes are TEMPO-oxidation with sonication and etherification with sonification at 1090 and 1030 kg CO_2_-eq/kg CNF, respectively. GHG emissions are primarily due to chemical and energy use for most processes, which are responsible for >90% of GHG emissions for all but three inventories (Figure 3). The exceptions to this are the three lowest GHG emission processes, where pulping makes up 15–23% of total GHG emissions, as a result of low energy and chemical related emissions. Relative to other process steps, emissions associated with the kraft pulping step are similar between inventories at an average of 1.2 ± 0.8 kg CO_2_-eq/kg nanocellulose. Similar trends in GHG emissions for each process are observed for other feedstocks (SI Table S25).

When we consider the production of CNCs and CNFs from five feedstocks—bleached kraft pulp, cotton linters, industrial waste, kraft pulp, and thermo-mechanical pulp—we see a slight variation in GHG emissions driven by the cellulose source selected. While the literature suggests pulp source can cause notable changes to environmental impacts of nanocellulose materials,^9^ a two-factor ANOVA considering the primary process and feedstock found no evidence of a difference in GHG emissions between feedstocks in this work (*p* = 1.00, SI Figure S1). This lack of impact is attributed to the relatively small shifts in GHG emissions between feedstocks relative to the substantial variation between alternative processes. However, for individual, low-emission processes, such as mechanical processing, meaningful shifts in impacts are observed between feedstocks (e.g., 1.8 kg CO_2_-eq/kg CNF for industrial waste vs 4.4 kg CO_2_-eq/kg CNF for cotton linters). While this study has insufficient data to determine if this difference is statistically significant, future work on reducing the GHG emissions of CNCs and CNFs should focus on the potential for both low-emission feedstocks and processes.

**Figure 4:**
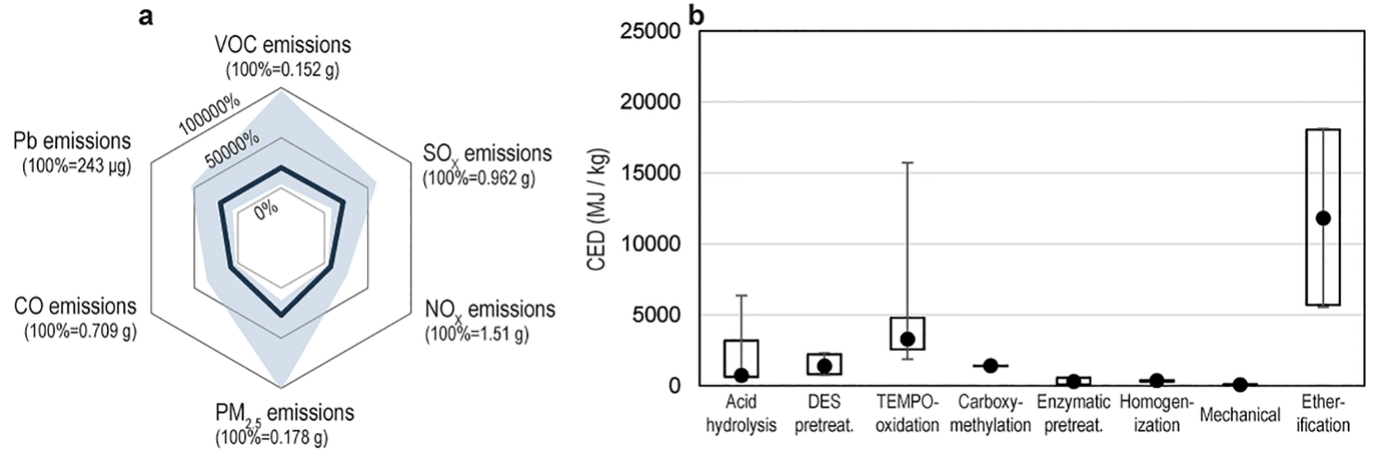
(a) Range of volatile organic compound (VOC) emissions, sulfur oxide (SO_X_) emissions, nitrogen oxide (NO_X_) emissions, particulate matter with diameter less than 2.5 μm (PM_2.5_) emissions, carbon monoxide (CO) emissions, and lead (Pb) emissions for the production of 1 kg CNCs and CNFs. The solid line represents the median, while the shaded region is the range of data between the 25^th^–75^th^ percentile. Data are normalized to the emissions of 1 kg of ordinary Portland cement as 100%. (b) Cumulative energy demand for each process examined to produce CNCs and CNFs. Here (●) represents the median, the upper and lower bounds of the boxes represent the 25^th^ and 75^th^ percentiles, and the upper and lower bounds of the whiskers represent the 5^th^ and 95^th^ percentiles. Note sample sizes differ between processes (SI Tables S26–S32).

Similar to findings for GHG emissions, broad ranges are observed for the other impact categories examined, namely CED, VOC emissions, SO_*x*_ emissions, NO_*x*_ emissions, PM_2.5_ emissions, CO emissions, and Pb emissions (Figure 4). As CED plays a crucial role in GHG emissions for most processes, similar trends in CED are seen as GHG emissions, with the lowest CED for the mechanical process (94 ± 38 MJ/kg CNF) and the highest for etherification (11 900 ± 6500 MJ/kg CNF). While performing a techno-economic analysis was outside the scope of this work, the high CED of some processes raises critical concerns regarding the cost of energy consumption for nanocellulose production. In January 2023, the US average electricity cost was 0.168 $/kWh.^32^ With this value, it can be estimated that the median cost associated with energy consumption alone ranges from $4.36/kg CNF using mechanical processes to $553/kg CNF using etherification.

Despite harmonization, a similar range of emissions is observed for the GHG emissions from the harmonized inventories (1.8–1100 kg CO_2_-eq/kg nanocellulose, median of 47.5 kg CO_2_-eq/kg nanocellulose) as the studies from which these inventories were derived (0.79–1160 kg CO_2_-eq/kg nanocellulose, median of 63 kg CO_2_-eq/kg nanocellulose). The lower median value of the harmonized inventory is considered to be primarily due to the removal of waste treatment from some inventories, when considering waste treatment, the median GHG emissions from the harmonized inventories rises to 74 kg CO2-eq/kg nanocellulose (SI Figure S3). Despite minimal change in the overall range, when directly comparing GHG emissions to their original studies with the same feedstock, the average process modeled had an increase of 44 kg CO_2_-eq/kg nanocellulose relative to the original study. For 16 out of 22 processes, harmonization increased GHG emissions relative to the original study. These findings suggest that the variation observed between past studies is primarily due to differences in the production process rather than methodology or assumptions. For example, when considering acid hydrolysis processes, the total mass of nonwater material inputs ranges between 25–83 kg/kg nanocellulose. As a result, the GHG emissions associated with chemical production vary from 5.5 to 155 kg CO_2_-eq/kg nanocellulose. Similarly, large ranges in CED are observed for many process types (Figure 4b). These key differences between underlying processes of a single process type highlight the wide variations in inputs observed throughout the harmonized inventories, which in turn drive the variation in environmental impacts observed.

This study did not consider carbon uptake in the biogenic resources used to produce nanocellulose. For most processes, the carbon uptake would have been small relative to the GHG emissions associated with CNC or CNF production. Assuming CNCs and CNFs to be purely cellulose, they have carbon uptake values of 1.63 kg CO_2_-eq/kg of nanocellulose. For low-emission CNC and CNF isolation processes (e.g., mechanical processing), including carbon uptake would result in up to a 90% reduction in cradle-to-gate GHG emissions. However, for an average across all processes and feedstocks, only a 0.9% reduction is found. Further, CNCs and CNFs may biodegrade at the end-of-life, which could lead to a release of biogenic carbon. We also note that this study did not consider the potential application of coproducts (e.g., kraft lignin from kraft pulping). As pulp typically was a minor contributor to GHG emissions (median contribution of 1.4% of total GHG emissions for kraft pulp), coproduct allocation would be expected to have little impact on emissions for nanocellulose for most of the processes considered. For low GHG emission processes, however, pulping plays a more meaningful role, contributing an average of 17.5% of GHG emissions for the three lowest GHG emission processes. In these cases, careful consideration of application of coproducts, as well as allocation method is needed to determine full process emissions.

**Figure 5:**
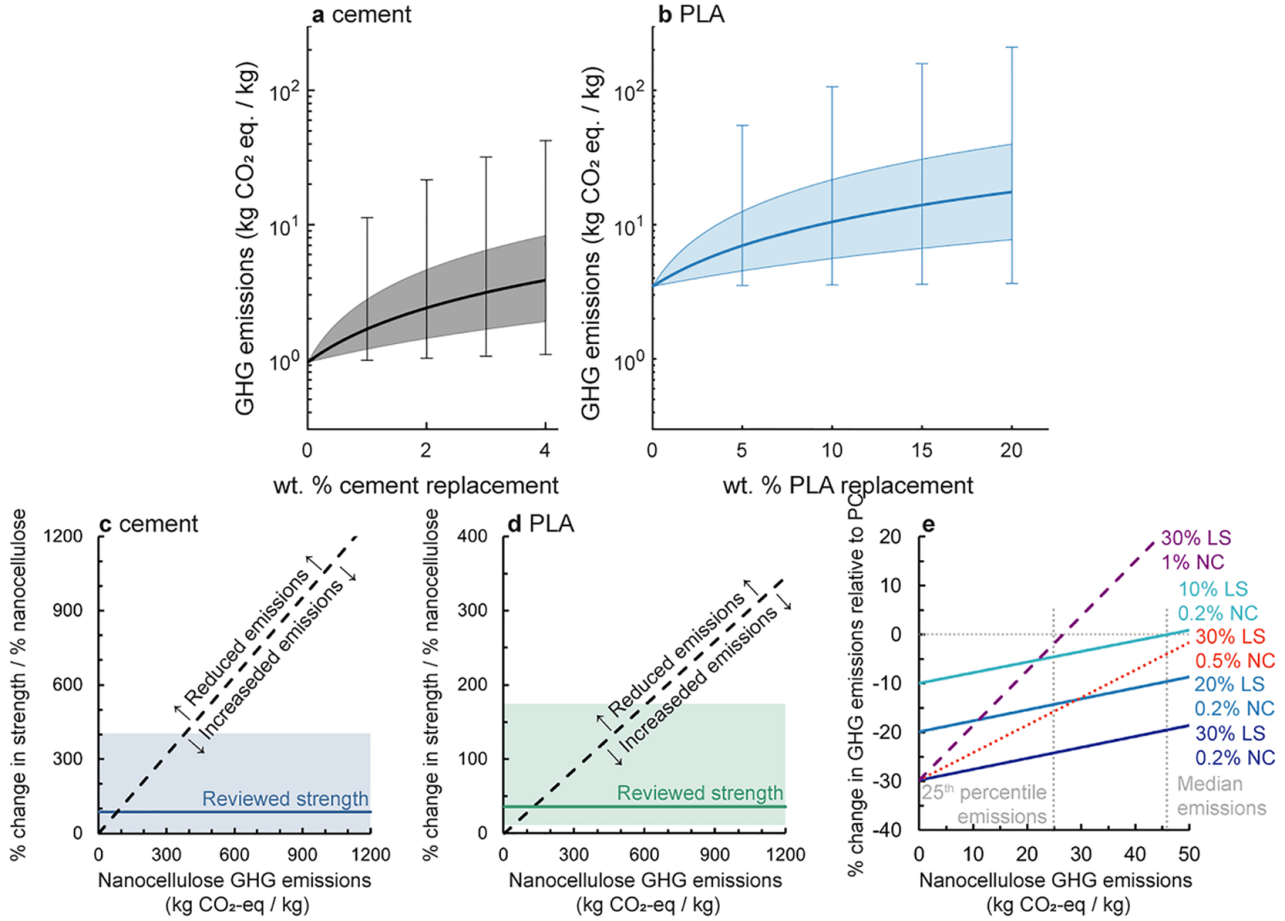
Effect of CNC or CNF addition on log-scale GHG emissions of (a) Portland cement and (b) PLA for typical replacement levels of each material.^10,30^ The solid line displays the median of nanocellulose GHG emissions, the shaded region is the range of data between the 25^th^–75^th^ percentile, and the upper and lower bounds of the whiskers represent the 5^th^ and 95^th^ percentiles. Required increase in strength to result in a decrease in GHG emissions for (c) cementitious composites on a compressive strength basis and (d) PLA composites on a tensile strength basis. The shaded region shows 5^th^–95^th^ percentile values and median values based on review studies for cementitious composites^35^ and PLA.^36^ The region above the dashed line represents the area where improved performance is sufficient to account for increased emissions. (e) Impact of replacement of Portland cement with limestone (LS) filler on GHG emissions of Portland cement composites with nanocellulose (NC) for a range of nanocellulose GHG emissions. 25^th^ percentile and median emissions for nanocellulose are labeled.

CNCs and CNFs have previously been considered environmentally sustainable additives to cementitious and polymer composites.^4,5^ However, when examining the impact of producing CNCs, CNFs, and Portland cement, we find the environmental impacts associated with the production of CNCs and CNFs are higher than the production of an equivalent mass of Portland cement across all impact categories. For example, with the inventories considered in this study, the use of the median GHG emissions for CNFs and replacement of 1 wt.% OPC with CNFs would increase GHG emissions by 45%. Again, considering a 1 wt % replacement, for the process with the least emissions (mechanical separation), GHG emissions would increase by 1% compared to a 1250% increase for the maximum emission process (etherification and sonication). The effects of weight replacement for cement nanocomposites are presented in Figure 5a.

Similarly, PLA production results in lower GHG emissions than 97% of the CNC or CNF production pathways considered in this study and typically lower emissions across other impact categories modeled. As a result, adding 10 wt % of the median nanocellulose process considered in this study to PLA would increase GHG emissions by over 110% relative to neat PLA (compared to a 5% reduction in GHG emissions for the lowest impact process and an over 3000% increase in emissions for the highest impact process).

For the addition of nanocellulose to be environmentally beneficial there are two main approaches. The first approach would rely on a gain in performance (e.g., mechanical strength) of cementitious or polymer composites with nanocellulose to offset the increased GHG emissions of nanocellulose. The second approach would rely on the nanocellulose to reduce the volume of an existing material with a GHG emission, like using cellulose and limestone to replace cement clinker.

To begin we can discuss the approach of improving a mechanical property. CNCs and CNFs have broadly been reported to potentially increase the strength and stiffness of cementitious composites.^10,35^ For cementitious composites, if a 4% increase in strength could be achieved per 1% nanocellulose addition, then there would be negligible change to GHG emissions per unit strength of the composite material at the 5^th^ percentile value for GHG emissions (Figure 5c). Strength increases greater than 4% would lead to a beneficial outcome (lower GHG emissions per unit strength of the composite material than if no nanocellulose was used). At the 25^th^ percentile value for GHG emissions, a 25% or greater increase in strength would be needed for such a beneficial outcome, and a 49% increase in strength would be needed at the median value for GHG emissions nanocellulose determined herein. When examining reviewed nanocellulose cementitious composite strengths, a median increase of 86% per % nanocellulose addition is observed, with a 95^th^ percentile value of a 411% increase per % nanocellulose addition. We note that often, additions of nanocellulose to cement are <1 wt % (e.g., a 54.7% increase in strength at 0.3 wt % nanocellulose addition would result in a XX% increase in strength in Figure 5c). In these cases, a lower absolute increase in strength is needed to reduce emissions. Nanocellulose may result in a reduction in GHG emissions of cementitious composites with a strength functional unit up to nanocellulose production GHG emissions of 82 kg CO_2_-eq/kg of nanocellulose at the median strength value, and 388 kg CO_2_-eq/kg of nanocellulose at the 95^th^ percentile value.

Similarly, PLA composites typically increase in strength with the addition of nanocellulose.^36^ However, due to the higher GHG emissions associated with PLA production, lower increases in strength are needed to reduce emissions on a strength basis than in cementitious composites. Only a 0.26% increase in strength per 1% nanocellulose addition is needed to reduce GHG emissions with a strength functional unit at the 5^th^ percentile value for nanocellulose GHG emissions, while only 6.2% increases and 12.7% increases are needed at the 25^th^ percentile and median nanocellulose emission values, respectively (Figure 5d). Nanocellulose production emissions of up to 128 kg CO_2_-eq/kg of nanocellulose at the median strength value reviewed from the literature, and 568 kg CO_2_-eq/kg of nanocellulose at the 95^th^ percentile strength value will still result in a reduction in total composite GHG emissions on a strength basis.

Targeting nanocellulose production processes that optimize for both low GHG emissions and high increases in strength per % nanocellulose addition result in the lowest emission outcomes on a strength basis. We note that these values are considered herein independently of nanocellulose characteristics, percent nanocellulose addition, other material properties, or composite processing (e.g., workability of concrete). Additionally, some applications of concrete may not utilize increased strength due to geometric or other design limitations. Future design and LCA of nanocellulose composites should holistically consider material application and all relevant properties to minimize emissions. Trade-offs in higher nanocellulose production emissions may be beneficial if that process produces higher quality nanocellulose that results in stronger composite materials, particularly for structural applications where increased strength may decrease the volume of required material.

To address the second approach, adding CNC or CNF to cementitious composites may allow for the replacement of Portland cement with low-carbon alternatives while maintaining sufficient strength. For example, Ramanathan et al. found that the addition of 0.2 vol % CNC to a cementitious composite of 70 wt % OPC and 30 wt % limestone resulted in comparable flexural strength to pure OPC, at a 29% reduction in GHG emissions relative to pure Portland cement.^37^ However, it should be noted that Ramanathan et al. considered CNCs to be net neutral in GHG emissions. When considering the range of GHG emissions for nanocellulose production determined herein, these limestone-cement-nanocellulose composites result in meaningful reductions in GHG emissions relative to pure OPC (Figure 5c, noting that we have converted from vol % of a nanocellulose slurry examined by Ramanathan et al. to wt % of nanocellulose). Due to the low emissions of limestone, with only limestone addition, GHG emissions are reduced by approximately the same percentage as the addition of limestone. As a result, when composites are modeled with the lowest quartile of nanocellulose emissions calculated herein results in a reduction in GHG emissions relative to OPC. For example, when 10 wt % OPC is replaced with limestone at 0.2 wt % limestone addition, GHG emissions are reduced relative to OPC until GHG emissions from nanocellulose production exceed 75 kg CO_2_-eq/kg. However, as the amount of nanocellulose added increases, reductions in GHG emissions are rapidly lost for high-emission nanocellulose production processes—at 30 wt % replacement with limestone and 1 wt % nanocellulose addition, GHG emissions relative to Portland cement are only reduced when emissions from nanocellulose production are less than 34 kg CO_2_-eq/kg. This scenario highlights how the addition of nanocellulose to cementitious composites can be applied in combination with other strategies to reduce GHG emissions of cementitious composites while maintaining or improving mechanical properties. When nanocellulose emissions are higher than the median value reported herein, this is challenging even with high additions of low-carbon materials, demonstrating the need for commercial production of nanocellulose with lower emissions than the median to reduce the GHG emissions of concrete.

This work has several limitations that should be expanded upon in future studies. This cradle-to-gate analysis did not consider the impacts of CNC or CNF addition on composite material lifespan as is a common approach used in LCA of cementitious materials. Despite this limitation, numerous studies have proposed that the addition of nanocellulose improves the durability of concrete, via decreased porosity and nanocellulose fibers bridging cracks.^33,34^ Effects of material durability on material life-cycle emissions is highly application-specific for both cementitious and plastic composites and beyond the scope of this cradle-to-gate analysis. However, in some cases increased durability could be expected to extend the usable service life of a product and therefore decrease life-cycle GHG emissions. Similarly to strength, an alternative approach could utilize reduced clinker content via nanocellulose and limestone addition, for an expected equivalent lifespan. For polymer composites, such as with PLA, potential improvements in durability with nanocellulose addition would be expected to play a more minor role, as durability is not the primary factor determining service life for many applications. In addition, this study did not consider the impact of each processing method on the quality and properties of the resulting CNC or CNF.

We note some inherent limitations of the inventories harmonized in this work that could contribute to the findings. All the inventories harmonized in this study were originally performed at the laboratory scale, except for the pilot scale inventories by Gu et al.^20^ and Sun et al.^21^ Not all of the processes we have studied in this work are currently being used at the commercial scale, and not all commercial scale processes are examined herein. For CNCs, acid hydrolysis is a common method, but TEMPO oxidation and enzymatic production pathways are predominantly still at the laboratory scale. For CNFs, purely mechanical pathways and TEMPO oxidation as a pretreatment followed by mechanical pathways are among the most prevalent methods. Laboratory scale processes are expected to have higher energy demand than pilot and commercial scale processes due to factors such as economies of scale. Therefore, in commercial CNCs and CNFs processes energy requirements could be expected to be lower. No complete LCA studies have previously compared lab and pilot-scale CNC and CNF isolation. However, past work has compared the energy consumption of pilot and lab-scale mechanical production of nanocellulose, finding a decrease of about 1 order of magnitude from lab to pilot scale.^38^ Notably, the lowest CED process herein (mechanical, 25.9 MJ/kg) was one of two pilot-scale processes examined, indicating potential for future reductions in CED as nanocellulose production scales up.

Treatment of wastewater, chemical waste, and biomass waste were excluded from this analysis, as complete waste flows were not reported for the majority of studies harmonized (Table 1). For studies where waste was reported, inclusion of waste treatment would typically play a minor role in overall emissions, contributing to <4% of total GHG emissions for all but two processes (SI Figure 2). For the processes reported by Zargar et al.^24^ and Gu et al.,^20^ waste treatment was responsible for 18.8% and 43.5% of total GHG emissions, respectively. Importantly, these were the only two acid hydrolysis processes to report complete waste treatment inventories, indicating that waste treatment may play a more important role in the GHG emissions of acid hydrolysis processes than in other process types, as a result of neutralizing sulfuric acid with sodium hydroxide.^20^

Herein, all emissions are allocated to the primary nanocellulose product. This aligns with the allocation method used by all but one of the studies examined. Gu et al. considered reduced impacts by offsetting primary production of materials with coproducts (e.g., sodium sulfate).^20^ Consideration of nanocellulose production as part of an entire biorefinery system and utilizing appropriate allocation or system expansion methods to assign environmental impacts to each product, will be an important factor in estimating environmental impacts of industrial scale nanocellulose production. Such consideration would be expected to reduce environmental impacts relative to the allocation method used herein. Processes can minimize their impacts by effectively utilizing biomass coproducts (e.g., kraft lignin from kraft pulping) and efficient recycling of chemicals used in the nanocellulose production process.

Despite past studies proposing nanocellulose as low environmental impact or carbon neutral, high GHG emissions were determined for many CNC and CNF isolation processes, highlighting the need for careful process design to limit GHG emissions. In this work, we harmonize 23 process designs and 115 combinations of process, process parameters, and feedstock to produce CNCs and CNFs. Our findings show significant variation in GHG emissions and other environmental impacts from producing CNCs and CNFs. Mechanical treatment was found to be the lowest GHG emission process to produce CNFs, and a sulfuric acid hydrolysis process to be the lowest GHG emission process for CNCs. For all processes considered in this study, GHG emissions to produce nanocellulose are higher than for Portland cement production, and most processes are higher than for PLA production. As a result, adding CNCs or CNFs to these materials is expected to increase cradle-to-gate GHG emissions. However, when GHG emissions are examined relative to material strength, increases in mechanical performance determined in past studies are sufficient to compensate for this increase for most nanocellulose production processes. In addition, other emissions examined, including SO_*x*_, NO_*x*_, and PM_2.5_ emissions, from most CNC and CNF production processes examined are higher than from producing Portland cement and PLA.

As many of the inventories harmonized in this study are at the laboratory scale, future work should examine larger-scale CNC and CNF production processes, which would be expected to result in reduced emissions. Future work on the environmental impacts of CNCs and CNFs should focus on low-emissions processes, such as mechanical separation and enzymatic hydrolysis, focusing on reducing process energy consumption. In addition, the impacts of CNCs and CNFs on cementitious and polymer composite durability and end-of-life should be examined to determine these full life cycle emissions and to address any aspects at subsequent life cycle stages that could alter findings.

The findings of this work highlight how biogenic feedstock does not unilaterally indicate low GHG emissions or an environmentally sustainable material. Despite the environmental challenges in CNC and CNF production for some processes, several pathways were noted with lower emissions, particularly by incorporation of nanocellulose into composite materials, where increased mechanical performance can offset the increased emissions from nanocellulose production. Advancing similar or improved low emission nanocellulose production pathways can best leverage the biogenic nature of these nanomaterials and their well-established ability to contribute to strong mechanical performance of nanocomposites.

## Supporting Information Available

Complete process descriptions (Table S1) and inventories (Tables S2–S24) for considered processes, comparison of nanocellulose GHG emissions by feedstock type (Figure S1), and tabulated results for all impact categories (Tables S25–S32) (PDF)
