# Supplementary material for: Harmonized Life-Cycle Inventories of Nanocellulose and Its Application in Composites
Source: Environ Sci Technol. 2023 Nov 15;57(48):19137–47. doi: 10.1021/acs.est.3c04814 (PMC10702438; doi:10.1021/acs.est.3c04814)
Supplement: Supplementary file 1 — es3c04814_si_001.pdf [file es3c04814_si_001.pdf]

Harmonized life-cycle inventories of nanocellulose and its application in composites

Seth Kane [a,†], Sabbie A. Miller [a], Kimberly E. Kurtis [b], Jeffrey Youngblood [c], Eric Landis [d], W. Jason Weiss [e]

[a] Department of Civil and Environmental Engineering, University of California, Davis
[b] School of Civil and Environmental Engineering, Georgia Institute of Technology
[c] School of Materials Engineering, Purdue University
[d] Department of Civil and Environmental Engineering, University of Maine
[e] School of Civil and Construction Engineering, Oregon State University

[†] Corresponding Author: skane@ucdavis.edu

This PDF file includes:
Supplementary Tables 1-32
Supplementary Figure 1-2
Supplementary References

Supplementary data and assumptions

Table S1. Summary of process steps modelled for the 23 process designs considered in this study.

**Cellulose**	**Reference**	**Key process**	**Original feedstock(s)**	**Process steps**
CNC	Gu et al.[1]	Acid hydrolysis	Bleached kraft pulp	Acid hydrolysis → Dilution → Bleaching → Neutralizing → Ultrafiltration → Concentration
CNC	Teh et al.[2]	Acid hydrolysis I	Empty fruit bunch	Washing → Wet milling → NaOH pretreatment → Bleaching → Washing → Acid hydrolysis → Dilution → Clarification→ Concentration→ Spray drying
		Acid hydrolysis II	Empty fruit bunch	Washing → Wet milling → Prehydrolysis → Soda pulping → Autoclave → Pressurizing → H_2O_2 bleaching → Washing → Acid hydrolysis → Dilution → Clarification → Concentration → Sonification → Spray drying
		TEMPO-oxidation	Empty fruit bunch	Washing → Wet milling → Prehydrolysis → Soda pulping → Autoclave → Pressurizing → H_2O_2 bleaching → Washing → Acid hydrolysis → Washing → TEMPO-oxidation with sonification Dilution → Clarification → Sonification → Clarification → Spray drying
CNC	de Figueirêdo et al.[3] *	Acid hydrolysis	White cotton fibers and coconut fibers	Chopping → Acid hydrolysis → Centrifugation → Washing
CNC	Zargar et al.[4]	Acid hydrolysis	Dissolving pulp	Acid hydrolysis → Dialysis → Centrifugation
		Deep eutectic solvent (DES) – minimum	Thermomechanical pulp	DES pretreatment → Dialysis → Blending → Centrifugation. Two rounds of DES treatment at 3.3 kg $C_5H_{14}ClNO$ / kg CNC and 1.6 kg ethanol / kg $C_5H_{14}ClNO$.
		Deep eutectic solvent (DES) – average	Thermomechanical pulp	DES pretreatment → Dialysis → Blending → Centrifugation. Three rounds of DES treatment at 5.5 kg $C_5H_{14}ClNO$ / kg CNC and 2.35 kg ethanol / kg $C_5H_{14}ClNO$.
		Deep eutectic solvent (DES) – maximum	Thermomechanical pulp	DES pretreatment → Dialysis → Blending → Centrifugation. Five rounds of DES treatment at 6.6 kg $C_5H_{14}ClNO$ / kg CNC and 3 kg ethanol / kg $C_5H_{14}ClNO$.

Table S1 (cont.). Summary of process steps modelled for the 23 process designs considered in this study.

CNF	Arvidsson et al.[5]	Enzymatic	Elementary Cl-free sulfate pulp, totally Cl-free sulfate pulp, unbleached sulfate pulp, and chlorine bleached sulphite pulp	Refining → Mixing → Incubation → Mixing → Washing → Enzyme denaturation → Washing → Refining → Adding microbicide → Microfluidization
		Carboxymethylation	Elementary Cl-free sulfate pulp, totally Cl-free sulfate pulp, unbleached sulfate pulp, and chlorine bleached sulphite pulp	Dispersion → Washing and filtration → Impregnation → Carboxymethylation → Washing and filtration → Impregnation → Washing and filtration → Microfluidization
		Homogenization	Elementary Cl-free sulfate pulp, totally Cl-free sulfate pulp, unbleached sulfate pulp, and chlorine bleached sulphite pulp	Homogenization
CNF	Stampino et al.[6]	Enzymatic	Cotton linter, kraft pulp, industrial waste sludge	Pre-refining → Enzymatic treatment → Refining → High pressure homogenization
		TEMPO-oxidation and homogenisation	Cotton linter, kraft pulp, industrial waste sludge	TEMPO-oxidation → Refining → High-pressure homongenisation
		TEMPO-oxidation and ultrasonication	Cotton linter, kraft pulp, industrial waste sludge	TEMPO-oxidation → ultrasonication
CNF	Li et al.[7]	Etherification and homogenization	Wood pulp	Chloroacetic acid etherification → Homogenization
		Etherification and sonication	Wood pulp	Chloroacetic acid etherification → Sonication → Centrifugation
		TEMPO-oxidation and homogenization	Wood pulp	TEMPO-oxidation → Homogenization
		TEMPO-oxidation and sonication	Wood pulp	TEMPO-oxidation → Sonication → Centrifugation
CNF	Moon et al.[8]	Mechanical – low energy	Wood pulp	Powdering → Wet cut milling → Hot compressed water treatment → Wet disk milling. Electricity use of 1.67 kWh / kg nanocellulose and heat use of 2.93 MJ / kg nanocellulose.
		Mechanical – high energy	Wood pulp	Powdering → Wet cut milling → Hot compressed water treatment → Wet disk milling. Electricity use of 5.05 kWh / kg nanocellulose and heat use of 6.84 MJ / kg nanocellulose.

* Note: these authors present inventories from cotton pulp and coconut fibers, the inventory for coconut was not utilized as it was more dissimilar from the pulp sources considered herein and did not lend itself to the homogenization efforts being performed.

Table S2. Inventory for 1kg of CNC - Acid hydrolysis - Forest Products Laboratory - kraft pulp (Gu et al.[1])

Resource	Quantity	Units	Notes
CNC	1	kg	Product
Materials/fuels			
Sulfate pulp {GLO}| market for | Cut-off, U	2.2244	kg	Bleached kraft dissolving pulp, feedstock (proxy)
Sulfuric acid {GLO}| market for | Cut-off, U	11.84	kg	Sulfuric acid, feedstock
Tap water {GLO}| market group for | Cut-off, U	229.4	kg	Reverse Osmosis water, dilution/bleaching/neutralization (modeled as tap water because electricity for reverse osmosis is reported), (units reported in liters, modeled as equivalent to kg)
Sodium hydroxide, without water, in 50% solution state {GLO}| market for | Cut-off, U	19.312	kg	Sodium Hydroxide NaOH, dilution/bleaching/neutralization (value increased to reflect 50% solution state)
Sodium hypochlorite, without water, in 15% solution state {GLO}| market for | Cut-off, U	0.267	kg	Sodium Chlorite NaClO2, dilution/bleaching/neutralization (proxy) (value increased to reflect 15% solution state)
Sodium hypochlorite, without water, in 15% solution state {GLO}| market for | Cut-off, U	0.0296	kg	Clorox Sodium Hypochloride, dilution/bleaching/neutralization (proxy) (value increased to reflect 15% solution state)
Tap water {GLO}| market group for | Cut-off, U	840	kg	Reverse Osmosis water, ultrafiltration (modeled as tap water because electricity for reverse osmosis is reported), (units reported in liters, modeled as equivalent to kg)
Electricity/heat			
Heat, from steam, in chemical industry {RoW}| market for heat, from steam, in chemical industry | Cut-off, U	0.55808	MJ	Steam, hydrolysis reaction (units given in kg, to convert to MJ, here the enthalpy of steam of 640 kJ/kg is used)
Electricity, low voltage {GLO}| market group for | Cut-off, U	0.13428	kWh	Electricity use in mixing, hydrolysis reaction
Electricity, low voltage {GLO}| market group for | Cut-off, U	0.4476	kWh	Electricity use for pump, hydrolysis reaction
Electricity, low voltage {GLO}| market group for | Cut-off, U	0.27976	kWh	Electricity use in mixing, dilution/bleaching/neutralization
Electricity, low voltage {GLO}| market group for | Cut-off, U	0.5728	kWh	Electricity use for Reverse Osmosis water, ultrafiltration
Electricity, low voltage {GLO}| market group for | Cut-off, U	10.7424	kWh	Electricity use for pump, ultrafiltration
Transport, lorry 3.5-16t, fleet average/US- US-EI U	0.44488	tkm	Assumed 200km of raw material transport
Waste to treatment*			
Waste paperboard {RoW}| market for waste paperboard | Cut-off, U	0.002	kg	waste (pulp waste), feedstock (proxy)
Molasses, from sugar beet {GLO}| market for | Conseq, U	1	kg	Sugar, hydrolysis reaction (proxy)
Treatment, fibre board production effluent, to wastewater treatment, class 3/US* US-EI U	0.008	l	ClO2, dilution/bleaching/neutralization (proxy) (units reported in kg, modeled here as equivalent to liters)
Treatment, fibre board production effluent, to wastewater treatment, class 3/US* US-EI U	1062.08	l	Waste water (proxy)
Treatment, fibre board production effluent, to wastewater treatment, class 3/US* US-EI U	0.01368	l	NaCl, ultrafiltration (proxy) (units reported in kg, modeled here as equivalent to liters)
Treatment, fibre board production effluent, to wastewater treatment, class 3/US* US-EI U	17.112	l	Na2SO4, ultrafiltration (proxy) (units reported in kg, modeled here as equivalent to liters)

*Waste to treatment is omitted from the analyses reported in text due to inconsistent reporting of waste flows in harmonized studies.

Table S3. Inventory for 1kg of CNC - Acid hydrolysis I - kraft pulp (Teh et al.[2])

Resource	Quantity	Units	Notes
CNC - Acid hydrolysis I - kraft pulp	1	kg	Product
Materials/fuels			
Sulfate pulp {GLO}| market for | Cut-off, U	1	kg	Pulp
Sodium hydroxide, without water, in 50% solution state {GLO}| market for | Cut-off, U	31.034	kg	NaOH, for Batch reactor (alkaline treatment) and Batch reactor (bleaching) (value increased to reflect 50% solution state)
Sulfuric acid {GLO}| market for | Cut-off, U	6.485	kg	H2SO4, for Batch reactor (acid hydrolysis)
Acetic acid, without water, in 98% solution state {GLO}| market for | Cut-off, U	18.238	kg	CH3COOH, for Batch reactor (bleaching)
Sodium hypochlorite, without water, in 15% solution state {GLO}| market for | Cut-off, U	26.247	kg	NaClO2, for Batch reactor (bleaching) (proxy) (value increased to reflect 15% solution state)
Water, deionised, from tap water, at user {RoW}| market for water, deionised, from tap water, at user | Cut-off, U	952.578	kg	Water, for all processes for which it is required (units given in liters, modeled as kg)
Electricity/heat			
Electricity, low voltage {GLO}| market group for | Cut-off, U	0.7227	MJ	Electricity, for all processes for which it is required
Heat, from steam, in chemical industry {RoW}| market for heat, from steam, in chemical industry | Cut-off, U	233.833	MJ	Thermal Energy, for all processes for which it is required
Transport, lorry 3.5-16t, fleet average/US- US-EI U	0.2	tkm	Assumed 200km of raw material transport

Table S4. Inventory for 1kg of CNC - Acid hydrolysis II - kraft pulp (Teh et al.[2])

Resource	Quantity	Units	Notes
CNC - Acid hydrolysis II - kraft pulp	1	kg	Product
Materials/fuels			
Sulfate pulp {GLO}| market for | Cut-off, U	1	kg	Pulp
Sodium hydroxide, without water, in 50% solution state {GLO}| market for | Cut-off, U	14.554	kg	NaOH, for Batch reactor (soda pulping) and Autoclave and Batch reactor (H2O2 bleaching) (value increased to reflect 50% solution state)
Magnesium sulfate {GLO}| market for | Cut-off, U	0.362	kg	MgSO4, for Autoclave and Batch reactor (H2O2 bleaching)
Oxygen, liquid {RoW}| market for | Cut-off, U	0.273	kg	O2, for Autoclave
Ozone, liquid {RoW}| market for | Cut-off, U	0.273	kg	O3, for Pressurizer
Hydrogen peroxide, without water, in 50% solution state {GLO}| market for | Cut-off, U	1.04	kg	H2O2, for Batch reactor (H2O2 bleaching) (value increased to reflect 50% solution state)
Sulfuric acid {GLO}| market for | Cut-off, U	9.571	kg	H2SO4, for Batch reactor (acid hydrolysis)
Water, deionised, from tap water, at user {RoW}| market for water, deionised, from tap water, at user | Cut-off, U	657.063	kg	Water, for all processes for which it is required (units given in liters, modeled as kg)
Electricity/heat			
Electricity, low voltage {GLO}| market group for | Cut-off, U	0.6242	MJ	Electricity, for all processes for which it is required
Heat, from steam, in chemical industry {RoW}| market for heat, from steam, in chemical industry | Cut-off, U	114.657	MJ	Thermal Energy, for all processes for which it is required
Transport, lorry 3.5-16t, fleet average/US- US-EI U	0.2	tkm	Assumed 200km of raw material transport

Table S5. Inventory for 1kg of CNC - Chemical acid hydrolysis - kraft pulp (de Figueirêdo et al.[3])

Resource	Quantity	Units	Notes
CNC - Chemical acid hydrolysis - kraft pulp	1	kg	Product
Materials/fuels			
Sulfate pulp {GLO}| market for | Cut-off, U	1.94	kg	Pulp
Sulfuric acid {GLO}| market for | Cut-off, U	23.29	kg	H2SO4
Water, deionised, from tap water, at user {RoW}| market for water, deionised, from tap water, at user | Cut-off, U	229.4	kg	water
Electricity/heat			
Electricity, low voltage {GLO}| market group for | Cut-off, U	500	kWh	Electricity use in mixing, hydrolysis reaction
Transport, lorry 3.5-16t, fleet average/US- US-EI U	0.388	tkm	Assumed 200km of raw material transport
Waste to treatment*			
Treatment, fibre board production effluent, to wastewater treatment, class 3/US* US-EI U	0.005	l	waste water (units reported in g, modeled here as equivalent to milliliters)

*Waste to treatment is omitted from the analyses reported in text due to inconsistent reporting of waste flows in harmonized studies.

Table S6. Inventory for 1kg of CNC - Sulfuric acid hydrolysis - kraft pulp (Zargar et al.[4])

Resource	Quantity	Units	Notes
CNC - Sulfuric acid hydrolysis - kraft pulp	1	kg	Product
Materials/fuels			
Sulfate pulp {GLO}| market for | Cut-off, U	3.333	kg	Pulp
Sulfuric acid {GLO}| market for | Cut-off, U	33.333	kg	98% H2SO4
Water, deionised, from tap water, at user {RoW}| market for water, deionised, from tap water, at user | Cut-off, U	33.333	kg	Dialysis water (units given in liters, modeled as kg)
Electricity/heat			
Electricity, low voltage {GLO}| market group for | Cut-off, U	0.4	kWh	Electricity for heating
Electricity, low voltage {GLO}| market group for | Cut-off, U	1	kWh	Electricity of centrifuge
Transport, lorry 3.5-16t, fleet average/US- US-EI U	0.667	tkm	Assumed 200km of raw material transport
Waste to treatment*			
Treatment, fibre board production effluent, to wastewater treatment, class 3/US* US-EI U	66.667	l	Remaining chemicals and water (units given in kg, modeled as l)
Waste paperboard {RoW}| market for waste paperboard | Cut-off, U	2.333	kg	Remaining raw materials

*Waste to treatment is omitted from the analyses reported in text due to inconsistent reporting of waste flows in harmonized studies.

Table S7. Inventory for 1kg of CNC - Lignin-containing - Deep eutectic solvent (DES) pretreatment, average - kraft pulp (Zargar et al.[4])

Resource	Quantity	Units	Notes
CNC - Lignin-containing - Deep eutectic solvent (DES) pretreatment, average - kraft pulp	1	kg	Product (inputs taken as an average from 11 studies by authors)
Materials/fuels			
Sulfate pulp {GLO}| market for | Cut-off, U	1.692	kg	Pulp
Chloroacetyl chloride {GLO}| market for | Cut-off, U	6.148	kg	Choline chloride (proxy)
Formic acid {RoW}| market for | Cut-off, U	4	kg	Oxalic acid (proxy)
Naphthalene sulfonic acid {GLO}| market for | Cut-off, U	3.955	kg	p-Toluenesulfonic acid (proxy)
Water, deionised, from tap water, at user {RoW}| market for water, deionised, from tap water, at user | Cut-off, U	16.923	kg	Dialysis water (units given in liters, modeled as kg)
Electricity/heat			
Electricity, low voltage {GLO}| market group for | Cut-off, U	16.985	kWh	Electricity for heating
Electricity, low voltage {GLO}| market group for | Cut-off, U	0.592	kWh	Electricity for blending
Electricity, low voltage {GLO}| market group for | Cut-off, U	0.508	kWh	Electricity of centrifuge
Heat, from steam, in chemical industry {RoW}| market for heat, from steam, in chemical industry | Cut-off, U	76.018	MJ	Energy for DES recovery
Transport, lorry 3.5-16t, fleet average/US- US-EI U	0.338	tkm	Assumed 200km of raw material transport
Waste to treatment*			
Treatment, fibre board production effluent, to wastewater treatment, class 3/US* US-EI U	31.02	l	Remaining chemicals and water (units given in kg, modeled as l)
Waste paperboard {RoW}| market for waste paperboard | Cut-off, U	0.692	kg	Remaining raw materials

*Waste to treatment is omitted from the analyses reported in text due to inconsistent reporting of waste flows in harmonized studies.

Table S8. Inventory for 1kg of CNC - Lignin-containing - Deep eutectic solvent (DES) pretreatment, maximum - kraft pulp (Zargar et al.[4])

Resource	Quantity	Units	Notes
CNC - Lignin-containing - Deep eutectic solvent (DES) pretreatment, max - kraft pulp	1	kg	Product (inputs taken as maximum values from 11 studies by authors)
Materials/fuels			
Sulfate pulp {GLO}| market for | Cut-off, U	1.887	kg	Pulp
Chloroacetyl chloride {GLO}| market for | Cut-off, U	8.774	kg	Choline chloride (proxy)
Formic acid {RoW}| market for | Cut-off, U	10.132	kg	Oxalic acid (proxy)
Naphthalene sulfonic acid {GLO}| market for | Cut-off, U	7.604	kg	p-Toluenesulfonic acid (proxy)
Water, deionised, from tap water, at user {RoW}| market for water, deionised, from tap water, at user | Cut-off, U	18.868	kg	Dialysis water (units given in liters, modeled as kg)
Electricity/heat			
Electricity, low voltage {GLO}| market group for | Cut-off, U	27.17	kWh	Electricity for heating
Electricity, low voltage {GLO}| market group for | Cut-off, U	0.66	kWh	Electricity for blending
Electricity, low voltage {GLO}| market group for | Cut-off, U	0.566	kWh	Electricity of centrifuge
Heat, from steam, in chemical industry {RoW}| market for heat, from steam, in chemical industry | Cut-off, U	84.755	MJ	Energy for DES recovery
Transport, lorry 3.5-16t, fleet average/US- US-EI U	0.377	tkm	Assumed 200km of raw material transport
Waste to treatment*			
Treatment, fibre board production effluent, to wastewater treatment, class 3/US* US-EI U	34.585	l	Remaining chemicals and water (units given in kg, modeled as l)
Waste paperboard {RoW}| market for waste paperboard | Cut-off, U	0.887	kg	Remaining raw materials

*Waste to treatment is omitted from the analyses reported in text due to inconsistent reporting of waste flows in harmonized studies.

Table S9. Inventory for 1kg of CNC - Lignin-containing - Deep eutectic solvent (DES) pretreatment, minimum - kraft pulp (Zargar et al.[4])

Resource	Quantity	Units	Notes
CNC - Lignin-containing - Deep eutectic solvent (DES) pretreatment, min - kraft pulp	1	kg	Product (inputs taken as minimum values from 11 studies by authors)
Materials/fuels			
Sulfate pulp {GLO}| market for | Cut-off, U	1.471	kg	Pulp
Chloroacetyl chloride {GLO}| market for | Cut-off, U	4.368	kg	Choline chloride (proxy)
Formic acid {RoW}| market for | Cut-off, U	1.956	kg	Oxalic acid (proxy)
Water, deionised, from tap water, at user {RoW}| market for water, deionised, from tap water, at user | Cut-off, U	14.706	kg	Dialysis water (units given in liters, modeled as kg)
Electricity/heat			
Electricity, low voltage {GLO}| market group for | Cut-off, U	7.059	kWh	Electricity for heating
Electricity, low voltage {GLO}| market group for | Cut-off, U	0.515	kWh	Electricity for blending
Electricity, low voltage {GLO}| market group for | Cut-off, U	0.441	kWh	Electricity of centrifuge
Heat, from steam, in chemical industry {RoW}| market for heat, from steam, in chemical industry | Cut-off, U	66.059	MJ	Energy for DES recovery
Transport, lorry 3.5-16t, fleet average/US- US-EI U	0.294	tkm	Assumed 200km of raw material transport
Waste to treatment*			
Treatment, fibre board production effluent, to wastewater treatment, class 3/US* US-EI U	26.956	l	Remaining chemicals and water (units given in kg, modeled as l)
Waste paperboard {RoW}| market for waste paperboard | Cut-off, U	0.471	kg	Remaining raw materials

*Waste to treatment is omitted from the analyses reported in text due to inconsistent reporting of waste flows in harmonized studies.

Table S10. Inventory for 1kg of CNC - TEMPO-oxidation - kraft pulp (Teh et al.[2])

Resource	Quantity	Units	Notes
CNC - TEMPO-oxidation - kraft pulp	1	kg	Product
Materials/fuels			
Sulfate pulp {GLO}| market for | Cut-off, U	1	kg	Pulp
Sodium hydroxide, without water, in 50% solution state {GLO}| market for | Cut-off, U	9.634	kg	NaOH, for Batch reactor (soda pulping) and Autoclave and Batch reactor (H2O2 bleaching) and Batch reactor (TEMPO + sonification) (value increased to reflect 50% solution state)
Magnesium sulfate {GLO}| market for | Cut-off, U	0.141	kg	MgSO4, for Autoclave and Batch reactor (H2O2 bleaching)
Oxygen, liquid {RoW}| market for | Cut-off, U	0.137	kg	O2, for Autoclave
Ozone, liquid {RoW}| market for | Cut-off, U	0.137	kg	O3, for Pressurizer
Hydrogen peroxide, without water, in 50% solution state {GLO}| market for | Cut-off, U	0.402	kg	H2O2, for Batch reactor (H2O2 bleaching) (value increased to reflect 50% solution state)
Hydrochloric acid, without water, in 30% solution state {RoW}| market for | Cut-off, U	13.857	kg	HCl, for Batch reactor (acid hydrolysis) and Batch reactor (TEMPO + sonification) (value increased to reflect 30% solution state)
Piperidine {GLO}| market for | Cut-off, U	0.019	kg	4-Acetamido-TEMPO, for Batch reactor (TEMPO + sonification) (proxy)
Ethylene bromide {GLO}| market for | Cut-off, U	0.189	kg	NaBr, for Batch reactor (TEMPO + sonification) (proxy)
Sodium hypochlorite, without water, in 15% solution state {GLO}| market for | Cut-off, U	87.54	kg	NaOCl, for Batch reactor (TEMPO + sonification) (value increased to reflect 15% solution state)
Ethanol, without water, in 95% solution state, from fermentation {GLO}| market for | Cut-off, U	9.334	kg	Ethanol, C2H5OH, for Batch reactor (TEMPO + sonification)
Water, deionised, from tap water, at user {RoW}| market for water, deionised, from tap water, at user | Cut-off, U	385.134	kg	Water, for all processes for which it is required (units given in liters, modeled as kg)
Electricity/heat			
Electricity, low voltage {GLO}| market group for | Cut-off, U	0.4344	MJ	Electricity, for all processes for which it is required
Heat, from steam, in chemical industry {RoW}| market for heat, from steam, in chemical industry | Cut-off, U	64.299	MJ	Thermal Energy, for all processes for which it is required
Transport, lorry 3.5-16t, fleet average/US- US-EI U	0.2	tkm	Assumed 200km of raw material transport

Table S11. Inventory for 1kg of CNF - Carboxymethylation pretreatments and microfluidization - kraft pulp (Arvidsson et al.[5])

Resource	Quantity	Units	Notes
CNF - Carboxymethylation pretreatments and microfluidization - kraft pulp	1	kg	Product
Materials/fuels			
Sulfate pulp {GLO}| market for | Cut-off, U	1	kg	Pulp
Water, deionised, from tap water, at user {RoW}| market for water, deionised, from tap water, at user | Cut-off, U	480	kg	Deionised water (units given in liters, modeled as kg)
Chloroacetic acid {GLO}| market for | Cut-off, U	0.09	kg	Monochloroacetic acid (proxy)
Isopropanol {GLO}| market for | Cut-off, U	18	kg	Isopropanol
Sodium hydroxide, without water, in 50% solution state {GLO}| market for | Cut-off, U	0.3	kg	Sodium hydroxide (0.15 kg)
Methanol {GLO}| market for | Cut-off, U	3.6	kg	Methanol
Acetic acid, without water, in 98% solution state {GLO}| market for | Cut-off, U	0.01	kg	Acetic acid
Sodium carbonate from ammonium chloride production, at plant/GLO US-EI U	0.76	kg	Sodium bicarbonated (proxy)
Electricity/heat			
Electricity, low voltage {GLO}| market group for | Cut-off, U	8	MJ	Electricity (microfluidization)
Heat, from steam, in chemical industry {RoW}| market for heat, from steam, in chemical industry | Cut-off, U	2.3	MJ	Heat
Transport, lorry 3.5-16t, fleet average/US- US-EI U	0.2	tkm	Assumed 200km of raw material transport

Table S12. Inventory for 1kg of CNF - Enzymatic pretreatments and microfluidization - kraft pulp (Arvidsson et al.[5])

Resource	Quantity	Units	Notes
CNF - Enzymatic pretreatments and microfluidization - kraft pulp	1	kg	Product
Materials/fuels			
Sulfate pulp {GLO}| market for | Cut-off, U	1	kg	Pulp
Water, deionised, from tap water, at user {RoW}| market for water, deionised, from tap water, at user | Cut-off, U	130	kg	Deionised water (units given in liters, modeled as kg)
Enzymes {GLO}| market for enzymes | Cut-off, U	0.00017	kg	enzymes
Sodium phosphate {GLO}| market for | Cut-off, U	0.042	kg	phosphate buffer (11 KH2PO4: 9 Na2HPO4) (proxy)
Biocides, for paper production, unspecified, at plant/US- US-EI U	0.01	kg	microbiocide
Electricity/heat			
Electricity, low voltage {GLO}| market group for | Cut-off, U	0.44	MJ	Electricity for refinement
Heat, from steam, in chemical industry {RoW}| market for heat, from steam, in chemical industry | Cut-off, U	9.6	MJ	Heat (proxy)
Electricity, low voltage {GLO}| market group for | Cut-off, U	8	MJ	electricity with pretreatment (microfluidization)
Transport, lorry 3.5-16t, fleet average/US- US-EI U	0.2	tkm	Assumed 200km of raw material transport

Table S13. Inventory for 1kg of CNF - Enzymatic treatments and homogenisation - kraft pulp (Stampino et al.[6])

Resource	Quantity	Units	Notes
CNF - Enzymatic treatments and homogenisation - kraft pulp	1	kg	Product
Materials/fuels			
Sulfate pulp {GLO}| market for | Cut-off, U	1.11	kg	Pulp
Water, deionised, from tap water, at user {RoW}| market for water, deionised, from tap water, at user | Cut-off, U	50	kg	Deionised water (units given in liters, modeled as kg)
Enzymes {GLO}| market for enzymes | Cut-off, U	0.13	g	FibreCare R (NovoEnzymes) (proxy)
Water, deionised, from tap water, at user {RoW}| market for water, deionised, from tap water, at user | Cut-off, U	50	kg	Deionised water (units given in liters, modeled as kg)
Tap water {GLO}| market group for | Cut-off, U	550	kg	Cooling water (units given in liters, modeled as kg)
Electricity/heat			
Electricity, low voltage {GLO}| market group for | Cut-off, U	0.46	kWh	Electricity (pulping)
Electricity, low voltage {GLO}| market group for | Cut-off, U	1.01	kWh	Electricity (pre-refining)
Electricity, low voltage {GLO}| market group for | Cut-off, U	8.31	kWh	Electricity (stirring)
Electricity, low voltage {GLO}| market group for | Cut-off, U	0.69	kWh	Electricity (pumping)
Electricity, low voltage {GLO}| market group for | Cut-off, U	1.01	kWh	Electricity (refining)
Electricity, low voltage {GLO}| market group for | Cut-off, U	30.47	kWh	Electricity (homogenisation)
Transport, lorry 3.5-16t, fleet average/US- US-EI U	0.222	tkm	Assumed 200km of raw material transport
Waste to treatment*			
Treatment, fibre board production effluent, to wastewater treatment, class 3/US* US-EI U	0.05	l	Waste water (proxy)

*Waste to treatment is omitted from the analyses reported in text due to inconsistent reporting of waste flows in harmonized studies.

Table S14. Inventory for 1kg of CNF - Etherification and homogenisation - kraft pulp (Li et al.[7])

Resource	Quantity	Units	Notes
CNF - Etherification and homogenisation - kraft pulp	1	kg	Product
Materials/fuels			
Sulfate pulp {GLO}| market for | Cut-off, U	4	kg	Pulp
Sodium hydroxide, without water, in 50% solution state {GLO}| market for | Cut-off, U	0.282	kg	NaOH (value increased to reflect 50% solution state)
Ethanol, without water, in 95% solution state, from fermentation {GLO}| market for | Cut-off, U	26.2	kg	Ethanol
Chloroacetic acid {GLO}| market for | Cut-off, U	0.212	kg	Chloroacetic acid
Isopropanol {GLO}| market for | Cut-off, U	43.5	kg	Isopropanol
Water, deionised, from tap water, at user {RoW}| market for water, deionised, from tap water, at user | Cut-off, U	12	kg	water (reaction) (units given in liters, modeled as kg)
Water, deionised, from tap water, at user {RoW}| market for water, deionised, from tap water, at user | Cut-off, U	500	kg	water (washing) (units given in liters, modeled as kg)
Electricity/heat			
Electricity, low voltage {GLO}| market group for | Cut-off, U	12	kWh	Electricity (heating and stirring plate)
Electricity, low voltage {GLO}| market group for | Cut-off, U	7	kWh	Electricity (centrifuge washer)
Electricity, low voltage {GLO}| market group for | Cut-off, U	96	kWh	Electricity (homogenizer)
Transport, lorry 3.5-16t, fleet average/US- US-EI U	0.8	tkm	Assumed 200km of raw material transport
Waste to treatment*			
Treatment, fibre board production effluent, to wastewater treatment, class 3/US* US-EI U	69.7	l	Waste water (proxy) (units given in grams, modeled as ml)

*Waste to treatment is omitted from the analyses reported in text due to inconsistent reporting of waste flows in harmonized studies.

Table S15. Inventory for 1kg of CNF - Etherification and sonification - kraft pulp (Li et al.[7])

Resource	Quantity	Units	Notes
CNF - Etherification and sonification - kraft pulp	1	kg	Product
Materials/fuels			
Sulfate pulp {GLO}| market for | Cut-off, U	4	kg	Pulp
Sodium hydroxide, without water, in 50% solution state {GLO}| market for | Cut-off, U	0.282	kg	NaOH (value increased to reflect 50% solution state)
Ethanol, without water, in 95% solution state, from fermentation {GLO}| market for | Cut-off, U	26.2	kg	Ethanol
Chloroacetic acid {GLO}| market for | Cut-off, U	0.212	kg	Chloroacetic acid
Isopropanol {GLO}| market for | Cut-off, U	43.5	kg	Isopropanol
Water, deionised, from tap water, at user {RoW}| market for water, deionised, from tap water, at user | Cut-off, U	12	kg	water (reaction) (units given in liters, modeled as kg)
Water, deionised, from tap water, at user {RoW}| market for water, deionised, from tap water, at user | Cut-off, U	500	kg	water (washing) (units given in liters, modeled as kg)
Electricity/heat			
Electricity, low voltage {GLO}| market group for | Cut-off, U	12	kWh	Electricity (heating and stirring plate)
Electricity, low voltage {GLO}| market group for | Cut-off, U	7	kWh	Electricity (centrifuge washer)
Electricity, low voltage {GLO}| market group for | Cut-off, U	330	kWh	Electricity (ultrasonication)
Electricity, low voltage {GLO}| market group for | Cut-off, U	785.4	kWh	Electricity (desktop centrifuge)
Transport, lorry 3.5-16t, fleet average/US- US-EI U	0.8	tkm	Assumed 200km of raw material transport
Waste to treatment*			
Treatment, fibre board production effluent, to wastewater treatment, class 3/US* US-EI U	69.7	1	Waste water (proxy) (units given in grams, modeled as ml)

*Waste to treatment is omitted from the analyses reported in text due to inconsistent reporting of waste flows in harmonized studies.

Table S16. Inventory for 1kg of CNF - Homogenisation, high energy - kraft pulp (Arvidsson et al.[5])

Resource	Quantity	Units	Notes
CNF - Homogenisation, high energy - kraft pulp	1	kg	Product
Materials/fuels			
Sulfate pulp {GLO}| market for | Cut-off, U	1	kg	Pulp
Electricity/heat			
Electricity, low voltage {GLO}| market group for | Cut-off, U	108	MJ	Electricity without pretreatment (homogenisation)
Transport, lorry 3.5-16t, fleet average/US- US-EI U	0.2	tkm	Assumed 200km of raw material transport

Table S17. Inventory for 1kg of CNF - Homogenisation, low energy - kraft pulp (Arvidsson et al.[5])

Resource	Quantity	Units	Notes
CNF - Homogenisation, low energy - kraft pulp	1	kg	Product
Materials/fuels			
Sulfate pulp {GLO}| market for | Cut-off, U	1	kg	Pulp
Electricity/heat			
Electricity, low voltage {GLO}| market group for | Cut-off, U	72	MJ	Electricity without pretreatment (homogenisation)
Transport, lorry 3.5-16t, fleet average/US- US-EI U	0.2	tkm	Assumed 200km of raw material transport

Table S18. Inventory for 1kg of CNF - Homogenisation, medium energy - kraft pulp (Arvidsson et al.[5])

Resource	Quantity	Units	Notes
CNF - Homogenisation, med energy - kraft pulp	1	kg	Product
Materials/fuels			
Sulfate pulp {GLO}| market for | Cut-off, U	1	kg	Pulp
Electricity/heat			
Electricity, low voltage {GLO}| market group for | Cut-off, U	97	MJ	Electricity without pretreatment (homogenisation)
Transport, lorry 3.5-16t, fleet average/US- US-EI U	0.2	tkm	Assumed 200km of raw material transport

Table S19. Inventory for 1kg of CNF - Mechanical, high energy - kraft pulp (Moon et al.[8])

Resource	Quantity	Units	Notes
CNF - Mechanical, high energy - kraft pulp	1	kg	Product
Materials/fuels			
Sulfate pulp {GLO}| market for | Cut-off, U	1	kg	pulp amount not specified (proxy)
Water, deionised, from tap water, at user {RoW}| market for water, deionised, from tap water, at user | Cut-off, U	19.44	kg	water (units provided in liters, modeled here in kg)
Electricity/heat			
Electricity, low voltage {GLO}| market group for | Cut-off, U	5.19	kWh	Power
Heat, from steam, in chemical industry {RoW}| market for heat, from steam, in chemical industry | Cut-off, U	7.11	MJ	Heat
Transport, lorry 3.5-16t, fleet average/US- US-EI U	0.2	tkm	Assumed 200km of raw material transport

Table S20. Inventory for 1kg of CNF - Mechanical, low energy - kraft pulp (Moon et al.[8])

Resource	Quantity	Units	Notes
CNF - Mechanical, low energy - kraft pulp	1	kg	Product
Materials/fuels			
Sulfate pulp {GLO}| market for | Cut-off, U	1	kg	pulp amount not specified (proxy)
Water, deionised, from tap water, at user {RoW}| market for water, deionised, from tap water, at user | Cut-off, U	17.84	kg	water (units provided in liters, modeled here in kg)
Electricity/heat			
Electricity, low voltage {GLO}| market group for | Cut-off, U	1.63	kWh	Power
Heat, from steam, in chemical industry {RoW}| market for heat, from steam, in chemical industry | Cut-off, U	2.83	MJ	Heat
Transport, lorry 3.5-16t, fleet average/US- US-EI U	0.2	tkm	Assumed 200km of raw material transport

Table S21. Inventory for 1kg of CNF - TEMPO-oxidation and homogenisation - kraft pulp (Stampino et al.[6])

Resource	Quantity	Units	Notes
CNF - TEMPO-oxidation and homogenisation - kraft pulp	1	kg	
Materials/fuels			
Sulfate pulp {GLO}| market for | Cut-off, U	1.06	kg	Pulp
Water, deionised, from tap water, at user {RoW}| market for water, deionised, from tap water, at user | Cut-off, U	60	kg	Deionised water (units given in liters, modeled as kg)
Potassium chloride, as K2O {GLO}| market for | Cut-off, U	0.16	kg	Potassium Bromide (proxy)
Piperidine {GLO}| market for | Cut-off, U	0.02	kg	TEMPO (proxy)
Sodium hypochlorite, without water, in 15% solution state {GLO}| market for | Cut-off, U	3.5	kg	NaClO (solution 10%) (units give in mL, modeled as g)
Sodium hydroxide, without water, in 50% solution state {GLO}| market for | Cut-off, U	0.04	kg	NaOH solution 4N (units give in mL, modeled as g)
Hydrochloric acid, without water, in 30% solution state {RER}| market for | Cut-off, U	0.04	kg	HCl solution 12N (units give in mL, modeled as g)
Tap water {GLO}| market group for | Cut-off, U	50	kg	Washing water (units given in liters, modeled as kg)
Water, deionised, from tap water, at user {RoW}| market for water, deionised, from tap water, at user | Cut-off, U	40	kg	Deionised water (units given in liters, modeled as kg)
Tap water {GLO}| market group for | Cut-off, U	440	kg	Cooling water (units given in liters, modeled as kg)
Tap water {GLO}| market group for | Cut-off, U	350	kg	Washing water (units given in liters, modeled as kg)
Electricity/heat			
Electricity, low voltage {GLO}| market group for | Cut-off, U	0.37	kWh	Electricity (pulping)
Electricity, low voltage {GLO}| market group for | Cut-off, U	119.42	kWh	Electricity (stirring)
Electricity, low voltage {GLO}| market group for | Cut-off, U	6.55	kWh	Electricity (pumping)
Electricity, low voltage {GLO}| market group for | Cut-off, U	0.81	kWh	Electricity (refining)
Electricity, low voltage {GLO}| market group for | Cut-off, U	9.52	kWh	Electricity (homogenisation)
Transport, lorry 3.5-16t, fleet average/US- US-EI U	0.212	tkm	Assumed 200km of raw material transport
Waste to treatment*			
Treatment, fibre board production effluent, to wastewater treatment, class 3/US* US-EI U	110	l	Waste water (proxy)

*Waste to treatment is omitted from the analyses reported in text due to inconsistent reporting of waste flows in harmonized studies.

Table S22. Inventory for 1kg of CNF - TEMPO-oxidation and homogenisation - kraft pulp (Li et al.[7])

**Resource**	**Quantity**	**Units**	**Notes**
CNF - TEMPO-oxidation and homogenisation - kraft pulp	1	kg	Product
Materials/fuels			
Sulfate pulp {GLO}| market for | Cut-off, U	4	kg	Pulp
Piperidine {GLO}| market for | Cut-off, U	0.006	kg	TEMPO (proxy)
Sodium hypochlorite, without water, in 15% solution state {GLO}| market for | Cut-off, U	12.853	kg	NaClO (value increased to reflect 15% solution state)
Ethylene bromide {GLO}| market for | Cut-off, U	0.24	kg	Sodium bromide (proxy)
Sodium hydroxide, without water, in 50% solution state {GLO}| market for | Cut-off, U	0.8	kg	NaOH (value increased to reflect 50% solution state)
Ethanol, without water, in 95% solution state, from fermentation {GLO}| market for | Cut-off, U	25	kg	Ethanol
Sodium chloride, powder {GLO}| market for | Cut-off, U	0.135	kg	Sodium chloride
Water, deionised, from tap water, at user {RoW}| market for water, deionised, from tap water, at user | Cut-off, U	50	kg	water (reaction) (units given in liters, modeled as kg)
Water, deionised, from tap water, at user {RoW}| market for water, deionised, from tap water, at user | Cut-off, U	500	kg	water (washing) (units given in liters, modeled as kg)
Electricity/heat			
Electricity, low voltage {GLO}| market group for | Cut-off, U	5.5	kWh	Electricity (cable blender)
Electricity, low voltage {GLO}| market group for | Cut-off, U	1.1	kWh	Electricity (syringe pump)
Electricity, low voltage {GLO}| market group for | Cut-off, U	7	kWh	Electricity (centrifuge washer)
Electricity, low voltage {GLO}| market group for | Cut-off, U	96	kWh	Electricity (homogenizer)
Transport, lorry 3.5-16t, fleet average/US- US-EI U	0.8	tkm	Assumed 200km of raw material transport
Waste to treatment*			
Treatment, fibre board production effluent, to wastewater treatment, class 3/US* US-EI U	25	l	Waste water (proxy) (units given in grams, modeled as ml)

*Waste to treatment is omitted from the analyses reported in text due to inconsistent reporting of waste flows in harmonized studies.

Table S23. Inventory for 1kg of CNF - TEMPO-oxidation and sonication - kraft pulp (Li et al.[7])

Resource	Quantity	Units	Notes
CNF - TEMPO-oxidation and sonication - kraft pulp	1	kg	Product
Materials/fuels			
Sulfate pulp {GLO}| market for | Cut-off, U	4	kg	Pulp
Piperidine {GLO}| market for | Cut-off, U	0.006	kg	TEMPO (proxy)
Sodium hypochlorite, without water, in 15% solution state {GLO}| market for | Cut-off, U	12.853	kg	NaClO (value increased to reflect 15% solution state)
Ethylene bromide {GLO}| market for | Cut-off, U	0.24	kg	Sodium bromide (proxy)
Sodium hydroxide, without water, in 50% solution state {GLO}| market for | Cut-off, U	0.8	kg	NaOH (value increased to reflect 50% solution state)
Ethanol, without water, in 95% solution state, from fermentation {GLO}| market for | Cut-off, U	25	kg	Ethanol
Sodium chloride, powder {GLO}| market for | Cut-off, U	0.135	kg	Sodium cloride
Water, deionised, from tap water, at user {RoW}| market for water, deionised, from tap water, at user | Cut-off, U	50	kg	water (reaction) (units given in liters, modeled as kg)
Water, deionised, from tap water, at user {RoW}| market for water, deionised, from tap water, at user | Cut-off, U	500	kg	water (washing) (units given in liters, modeled as kg)
Electricity/heat			
Electricity, low voltage {GLO}| market group for | Cut-off, U	5.5	kWh	Electricity (cable blender)
Electricity, low voltage {GLO}| market group for | Cut-off, U	1.1	kWh	Electricity (syringe pump)
Electricity, low voltage {GLO}| market group for | Cut-off, U	7	kWh	Electricity (centrifuge washer)
Electricity, low voltage {GLO}| market group for | Cut-off, U	330	kWh	Electricity (ultrasonication)
Electricity, low voltage {GLO}| market group for | Cut-off, U	785.4	kWh	Electricity (desktop centrifuge)
Transport, lorry 3.5-16t, fleet average/US- US-EI U	0.8	tkm	Assumed 200km of raw material transport
Waste to treatment*			
Treatment, fibre board production effluent, to wastewater treatment, class 3/US* US-EI U	25	l	Waste water (proxy) (units given in grams, modeled as ml)

*Waste to treatment is omitted from the analyses reported in text due to inconsistent reporting of waste flows in harmonized studies.

Table S24. Inventory for 1kg of CNF - TEMPO-oxidation and ultrasonication - kraft pulp (Stampino et al.[6])

Resource	Quantity	Units	Notes
CNF - TEMPO-oxidation and ultrasonication - kraft pulp	1	kg	Product
Materials/fuels			
Sulfate pulp {GLO}| market for | Cut-off, U	1.29	kg	Pulp
Water, deionised, from tap water, at user {RoW}| market for water, deionised, from tap water, at user | Cut-off, U	70	kg	Deionised water
Potassium chloride, as K2O {GLO}| market for | Cut-off, U	0.2	kg	Potassium Bromide (proxy)
Piperidine {GLO}| market for | Cut-off, U	0.03	kg	TEMPO (proxy)
Sodium hypochlorite, without water, in 15% solution state {GLO}| market for | Cut-off, U	4.3	kg	NaClO (solution 10%)
Sodium hydroxide, without water, in 50% solution state {GLO}| market for | Cut-off, U	0.39	kg	NaOH solution 4N
Hydrochloric acid, without water, in 30% solution state {RER}| market for | Cut-off, U	0.26	kg	HCl solution 12N
Tap water {GLO}| market group for | Cut-off, U	60	kg	Washing water
Sodium hydroxide, without water, in 50% solution state {GLO}| market for | Cut-off, U	0.05	kg	NaOH seeds
Water, deionised, from tap water, at user {RoW}| market for water, deionised, from tap water, at user | Cut-off, U	50	kg	Deionised water
Tap water {GLO}| market group for | Cut-off, U	130	kg	Cooling water
Electricity/heat			
Electricity, low voltage {GLO}| market group for | Cut-off, U	0.54	kWh	Electricity (pulping)
Electricity, low voltage {GLO}| market group for | Cut-off, U	173.97	kWh	Electricity (stirring)
Electricity, low voltage {GLO}| market group for | Cut-off, U	9.54	kWh	Electricity (pumping)
Electricity, low voltage {GLO}| market group for | Cut-off, U	6.9	kWh	Electricity (sonication)
Transport, lorry 3.5-16t, fleet average/US- US-EI U	0.258	tkm	Assumed 200km of raw material transport
Waste to treatment*			
Treatment, fibre board production effluent, to wastewater treatment, class 3/US* US-EI U	140	l	Waste water (proxy)

*Waste to treatment is omitted from the analyses reported in text due to inconsistent reporting of waste flows in harmonized studies.

Table S25. GHG emissions (in kg CO_2-eq / kg nanocellulose) for the 115 combinations of feedstock and process modeled.

Process study	Cellulose	Process	Bleached kraft pulp	Cotton Liners	Industrial Waste	Kraft pulp	Thermo-mechanical pulp
Gu et al.[1]	CNC	Acid hydrolysis	43.6	46.1	40.3	41.7	44.2
Teh et al.[2]	CNC	Acid hydrolysis I	186.1	187.2	184.6	185.2	186.3
Teh et al.[2]	CNC	Acid hydrolysis II	44.2	45.4	42.8	43.4	44.5
de Figueiredo et al.[3]	CNC	Acid hydrolysis	427.4	429.7	424.6	425.8	428.0
Zagar et al.[4]	CNC	Acid hydrolysis	11.7	15.5	6.8	8.9	12.6
Zagar et al.[4]	CNC	DES pretreatment - minimum	44.0	45.7	41.9	42.8	44.4
Zagar et al.[4]	CNC	DES pretreatment - average	75.3	77.3	72.8	73.9	75.8
Zagar et al.[4]	CNC	DES pretreatment - maximum	123.4	125.6	120.7	121.8	123.9
Teh et al.[2]	CNC	TEMPO-oxidation	314.1	315.3	312.7	313.3	314.4
Stampino et al.[6]	CNF	TEMPO-oxidation and homogenisation	127.8	129.0	153.6	126.9	128.1
Stampino et al.[6]	CNF	TEMPO-oxidation and ultrasonication	176.6	178.1	178.0	175.6	177.0
Li et al.[7]	CNF	TEMPO-oxidation and homogenisation	174.7	179.3	168.9	171.3	175.8
Li et al.[7]	CNF	TEMPO-oxidation and sonication	1031.7	1036.2	1025.8	1028.3	1032.8
Arvidsson et al.[5]	CNF	Carboxymethylation	47.5	48.6	46.0	46.6	47.7
Arvidsson et al.[5]	CNF	Enzymatic	5.1	6.3	3.6	4.3	5.4
Stampino et al.[6]	CNF	Enzymatic	37.4	38.7	34.9	36.5	37.7
Li et al.[7]	CNF	Etherification and homogenisation	235.0	239.6	229.2	231.7	236.1
Li et al.[7]	CNF	Etherification and sonification	1092.0	1096.6	1086.1	1088.6	1093.1
Arvidsson et al.[5]	CNF	Homogenization - low energy	18.3	19.5	16.9	17.5	18.6
Arvidsson et al.[5]	CNF	Homogenization - medium energy	24.2	25.3	22.7	23.3	24.4
Arvidsson et al.[5]	CNF	Homogenization - high energy	26.7	27.9	25.3	25.9	27.0
Sun et al.[8]	CNF	Mechanical - high energy	6.8	8.0	5.3	6.0	7.1
Sun et al.[8]	CNF	Mechanical - low energy	3.3	4.4	1.8	2.4	3.5

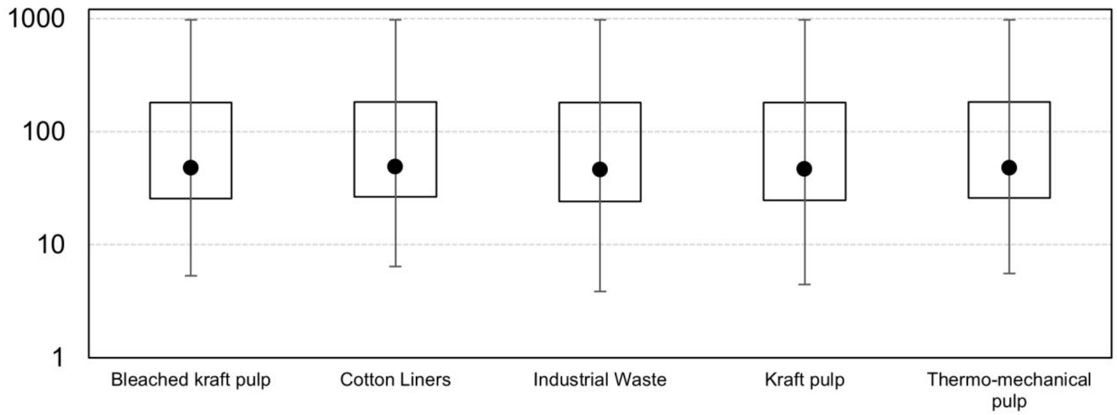

Supplemental Figure 1. Comparison of log-scale GHG emissions by feedstock for all 115 combinations of process and feedstock modelled. Here (•) represents the median, the upper and lower bounds of the boxes represent the 25th and 75th percentiles, and the upper and lower bounds of the whiskers represent the 5th and 95th percentiles.

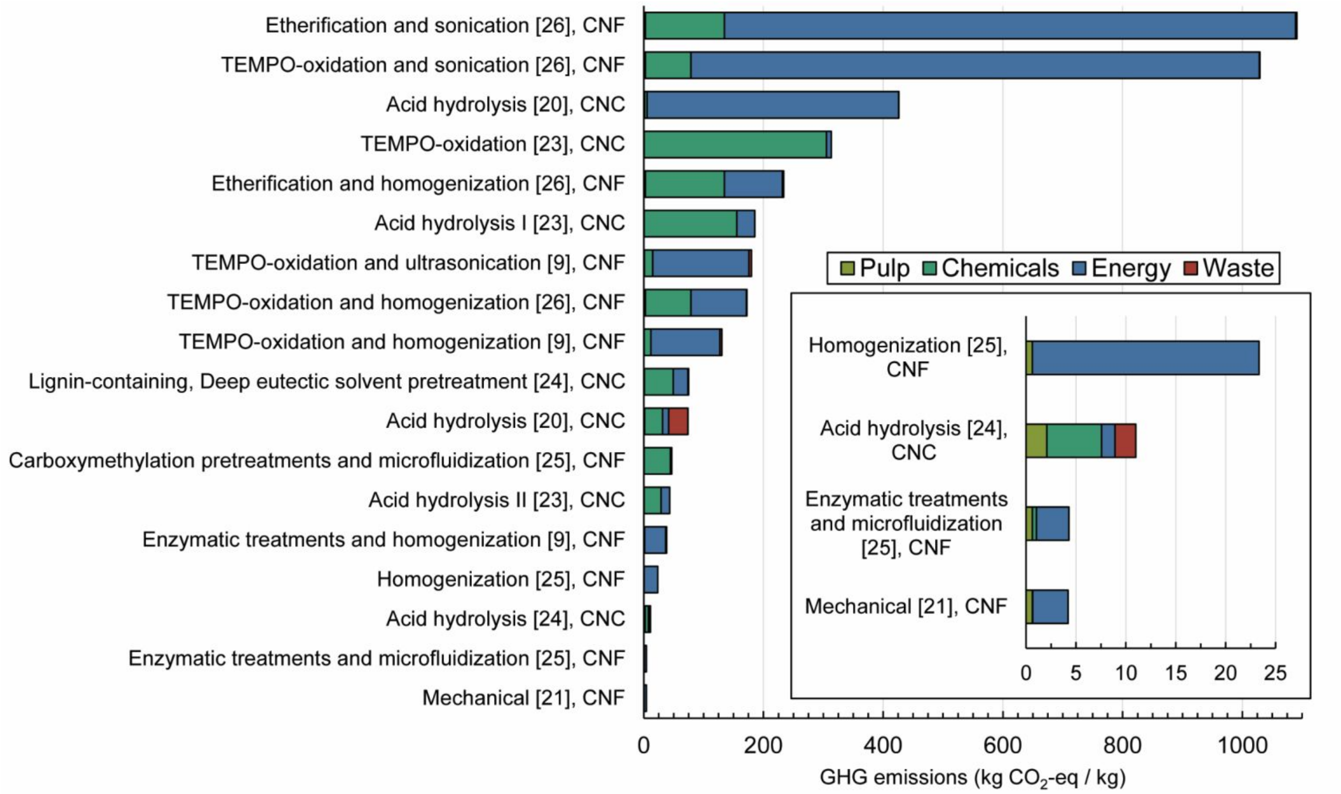

Supplemental Figure 2. Summary of inventory sources and greenhouse gas emissions associated with producing CNCs and CNFs using kraft pulp as the cellulose source, including treatment of waste for process for which it is available. For processes with multiple parameters examined, mean values are shown. Inset shows emissions for inventories < 25 kg CO_2-eq / kg in detail.

Table S26. CED (in MJ / kg nanocellulose) for the 115 combinations of feedstock and process modeled.

Process study	Cellulose	Process	Bleached kraft pulp	Cotton Liners	Industrial Waste	Kraft pulp	Thermo-mechanical pulp
Gu et al.[1]	CNC	Acid hydrolysis	771.7	792.6	635.5	747.3	705.5
Teh et al.[2]	CNC	Acid hydrolysis I	3196.5	3205.9	3135.3	3185.5	3166.7
Teh et al.[2]	CNC	Acid hydrolysis II	705.3	714.7	644.1	694.4	675.6
de Figueiredo et al.[3]	CNC	Acid hydrolysis	6362.9	6381.1	6244.1	6341.6	6305.2
Zagar et al.[4]	CNC	Acid hydrolysis	454.8	486.2	250.9	418.3	355.7
Zagar et al.[4]	CNC	DES pretreatment - minimum	823.3	837.1	733.3	807.2	779.5
Zagar et al.[4]	CNC	DES pretreatment - average	1411.6	1427.5	1308.0	1393.0	1361.2
Zagar et al.[4]	CNC	DES pretreatment - maximum	2317.7	2335.4	2202.2	2297.0	2261.5
Teh et al.[2]	CNC	TEMPO-oxidation	4787.1	4796.5	4725.9	4776.2	4757.4
Stampino et al.[6]	CNF	TEMPO-oxidation and homogenisation	1891.0	1900.9	2221.4	1879.4	1859.4
Stampino et al.[6]	CNF	TEMPO-oxidation and ultrasonication	2607.3	2619.5	2572.6	2593.2	2569.0
Li et al.[7]	CNF	TEMPO-oxidation and homogenisation	3347.7	3385.3	3102.9	3303.9	3228.7
Li et al.[7]	CNF	TEMPO-oxidation and sonication	15736.4	15774.0	15491.6	15692.6	15617.4
Arvidsson et al.[5]	CNF	Carboxymethylation	1428.1	1437.5	1366.9	1417.1	1398.3
Arvidsson et al.[5]	CNF	Enzymatic	112.6	122.0	51.4	101.6	82.8
Stampino et al.[6]	CNF	Enzymatic	585.9	596.3	505.4	573.7	552.8
Li et al.[7]	CNF	Etherification and homogenisation	5730.3	5767.9	5485.5	5686.5	5611.3
Li et al.[7]	CNF	Etherification and sonification	18119.0	18156.6	17874.2	18075.2	18000.0
Arvidsson et al.[5]	CNF	Homogenization - low energy	305.2	314.6	244.0	294.2	275.4
Arvidsson et al.[5]	CNF	Homogenization - medium energy	389.6	399.0	328.4	378.6	359.8
Arvidsson et al.[5]	CNF	Homogenization - high energy	426.7	436.1	365.5	415.7	397.0
Sun et al.[8]	CNF	Mechanical - high energy	137.7	147.1	76.5	126.7	107.9
Sun et al.[8]	CNF	Mechanical - low energy	87.1	96.5	25.9	76.2	57.4

Table S27. VOC emissions (in kg VOC / kg nanocellulose) for the 115 combinations of feedstock and process modeled.

Process study	Cellulose	Process	Bleached kraft pulp	Cotton Liners	Industrial Waste	Kraft pulp	Thermo-mechanical pulp
Gu et al.[1]	CNC	Acid hydrolysis	0.013	0.015	0.012	0.013	0.012
Teh et al.[2]	CNC	Acid hydrolysis I	0.187	0.188	0.187	0.187	0.187
Teh et al.[2]	CNC	Acid hydrolysis II	0.015	0.015	0.014	0.014	0.014
de Figueiredo et al.[3]	CNC	Acid hydrolysis	0.072	0.073	0.070	0.071	0.071
Zagar et al.[4]	CNC	Acid hydrolysis	0.011	0.013	0.008	0.010	0.010
Zagar et al.[4]	CNC	DES pretreatment - minimum	0.044	0.045	0.042	0.043	0.043
Zagar et al.[4]	CNC	DES pretreatment - average	0.148	0.149	0.147	0.148	0.147
Zagar et al.[4]	CNC	DES pretreatment - maximum	0.268	0.269	0.266	0.267	0.267
Teh et al.[2]	CNC	TEMPO-oxidation	0.086	0.087	0.085	0.086	0.086
Stampino et al.[6]	CNF	TEMPO-oxidation and homogenisation	0.022	0.023	0.027	0.022	0.022
Stampino et al.[6]	CNF	TEMPO-oxidation and ultrasonication	0.031	0.032	0.031	0.030	0.030
Li et al.[7]	CNF	TEMPO-oxidation and homogenisation	0.054	0.057	0.051	0.053	0.053
Li et al.[7]	CNF	TEMPO-oxidation and sonication	0.186	0.189	0.183	0.185	0.184
Arvidsson et al.[5]	CNF	Carboxymethylation	0.094	0.094	0.093	0.093	0.093
Arvidsson et al.[5]	CNF	Enzymatic	0.003	0.004	0.002	0.003	0.003
Stampino et al.[6]	CNF	Enzymatic	0.007	0.008	0.006	0.006	0.006
Li et al.[7]	CNF	Etherification and homogenisation	0.195	0.198	0.192	0.194	0.194
Li et al.[7]	CNF	Etherification and sonification	0.327	0.330	0.324	0.326	0.325
Arvidsson et al.[5]	CNF	Homogenization - low energy	0.004	0.004	0.003	0.003	0.003
Arvidsson et al.[5]	CNF	Homogenization - medium energy	0.005	0.005	0.004	0.004	0.004
Arvidsson et al.[5]	CNF	Homogenization - high energy	0.005	0.006	0.004	0.005	0.005
Sun et al.[8]	CNF	Mechanical - high energy	0.002	0.003	0.001	0.002	0.002
Sun et al.[8]	CNF	Mechanical - low energy	0.001	0.002	0.001	0.001	0.001

Table S28. SO_x emissions (in kg SO_2-eq / kg nanocellulose) for the 115 combinations of feedstock and process modeled.

Process study	Cellulose	Process	Bleached kraft pulp	Cotton Liners	Industrial Waste	Kraft pulp	Thermo-mechanical pulp
Gu et al.[1]	CNC	Acid hydrolysis	0.202	0.192	0.180	0.186	0.191
Teh et al.[2]	CNC	Acid hydrolysis I	0.587	0.583	0.577	0.580	0.582
Teh et al.[2]	CNC	Acid hydrolysis II	0.176	0.172	0.166	0.169	0.171
de Figueiredo et al.[3]	CNC	Acid hydrolysis	1.346	1.337	1.327	1.332	1.337
Zagar et al.[4]	CNC	Acid hydrolysis	0.223	0.208	0.190	0.199	0.206
Zagar et al.[4]	CNC	DES pretreatment - minimum	0.211	0.205	0.197	0.200	0.204
Zagar et al.[4]	CNC	DES pretreatment - average	0.351	0.343	0.334	0.339	0.343
Zagar et al.[4]	CNC	DES pretreatment - maximum	0.560	0.551	0.541	0.546	0.551
Teh et al.[2]	CNC	TEMPO-oxidation	0.966	0.962	0.956	0.959	0.961
Stampino et al.[6]	CNF	TEMPO-oxidation and homogenisation	0.372	0.367	0.442	0.364	0.367
Stampino et al.[6]	CNF	TEMPO-oxidation and ultrasonication	0.513	0.507	0.512	0.504	0.507
Li et al.[7]	CNF	TEMPO-oxidation and homogenisation	0.499	0.480	0.459	0.469	0.479
Li et al.[7]	CNF	TEMPO-oxidation and sonication	2.936	2.918	2.897	2.907	2.916
Arvidsson et al.[5]	CNF	Carboxymethylation	0.119	0.115	0.109	0.112	0.114
Arvidsson et al.[5]	CNF	Enzymatic	0.020	0.016	0.010	0.013	0.015
Stampino et al.[6]	CNF	Enzymatic	0.113	0.108	0.099	0.105	0.107
Li et al.[7]	CNF	Etherification and homogenisation	0.614	0.596	0.575	0.585	0.594
Li et al.[7]	CNF	Etherification and sonification	3.052	3.033	3.012	3.022	3.032
Arvidsson et al.[5]	CNF	Homogenization - low energy	0.058	0.053	0.048	0.050	0.053
Arvidsson et al.[5]	CNF	Homogenization - medium energy	0.074	0.070	0.065	0.067	0.069
Arvidsson et al.[5]	CNF	Homogenization - high energy	0.082	0.077	0.072	0.074	0.077
Sun et al.[8]	CNF	Mechanical - high energy	0.025	0.020	0.015	0.017	0.020
Sun et al.[8]	CNF	Mechanical - low energy	0.015	0.010	0.005	0.007	0.010

Table S29. NO_x emissions (in kg NO_2-eq / kg nanocellulose) for the 115 combinations of feedstock and process modeled.

Process study	Cellulose	Process	Bleached kraft pulp	Cotton Liners	Industrial Waste	Kraft pulp	Thermo-mechanical pulp
Gu et al.[1]	CNC	Acid hydrolysis	0.106	0.116	0.096	0.102	0.107
Teh et al.[2]	CNC	Acid hydrolysis I	0.383	0.387	0.378	0.381	0.383
Teh et al.[2]	CNC	Acid hydrolysis II	0.090	0.094	0.085	0.088	0.090
de Figueiredo et al.[3]	CNC	Acid hydrolysis	0.837	0.845	0.828	0.834	0.838
Zagar et al.[4]	CNC	Acid hydrolysis	0.055	0.069	0.039	0.049	0.056
Zagar et al.[4]	CNC	DES pretreatment - minimum	0.087	0.094	0.080	0.085	0.088
Zagar et al.[4]	CNC	DES pretreatment - average	0.150	0.158	0.143	0.148	0.151
Zagar et al.[4]	CNC	DES pretreatment - maximum	0.247	0.255	0.238	0.244	0.247
Teh et al.[2]	CNC	TEMPO-oxidation	0.691	0.696	0.687	0.690	0.692
Stampino et al.[6]	CNF	TEMPO-oxidation and homogenisation	0.250	0.254	0.300	0.248	0.250
Stampino et al.[6]	CNF	TEMPO-oxidation and ultrasonication	0.344	0.350	0.348	0.342	0.344
Li et al.[7]	CNF	TEMPO-oxidation and homogenisation	0.365	0.383	0.347	0.359	0.366
Li et al.[7]	CNF	TEMPO-oxidation and sonication	2.000	2.017	1.981	1.993	2.001
Arvidsson et al.[5]	CNF	Carboxymethylation	0.079	0.083	0.074	0.077	0.079
Arvidsson et al.[5]	CNF	Enzymatic	0.011	0.015	0.006	0.009	0.011
Stampino et al.[6]	CNF	Enzymatic	0.074	0.079	0.067	0.072	0.074
Li et al.[7]	CNF	Etherification and homogenisation	0.437	0.454	0.418	0.430	0.438
Li et al.[7]	CNF	Etherification and sonification	2.071	2.088	2.052	2.064	2.072
Arvidsson et al.[5]	CNF	Homogenization - low energy	0.037	0.041	0.032	0.035	0.037
Arvidsson et al.[5]	CNF	Homogenization - medium energy	0.048	0.053	0.044	0.047	0.049
Arvidsson et al.[5]	CNF	Homogenization - high energy	0.053	0.058	0.049	0.051	0.053
Sun et al.[8]	CNF	Mechanical - high energy	0.015	0.019	0.010	0.013	0.015
Sun et al.[8]	CNF	Mechanical - low energy	0.008	0.013	0.004	0.006	0.008

Table S30. $PM_{2.5}$ emissions (in kg $PM_{2.5}$-eq / kg nanocellulose) for the 115 combinations of feedstock and process modeled.

Process study	Cellulose	Process	Bleached kraft pulp	Cotton Liners	Industrial Waste	Kraft pulp	Thermo-mechanical pulp
Gu et al.[1]	CNC	Acid hydrolysis	0.046	0.048	0.045	0.048	0.049
Teh et al.[2]	CNC	Acid hydrolysis I	0.163	0.165	0.163	0.164	0.165
Teh et al.[2]	CNC	Acid hydrolysis II	0.034	0.035	0.034	0.035	0.036
de Figueiredo et al.[3]	CNC	Acid hydrolysis	0.503	0.505	0.502	0.505	0.505
Zagar et al.[4]	CNC	Acid hydrolysis	0.007	0.011	0.006	0.011	0.012
Zagar et al.[4]	CNC	DES pretreatment - minimum	0.033	0.035	0.033	0.035	0.035
Zagar et al.[4]	CNC	DES pretreatment - average	0.065	0.067	0.065	0.067	0.068
Zagar et al.[4]	CNC	DES pretreatment - maximum	0.110	0.112	0.109	0.112	0.112
Teh et al.[2]	CNC	TEMPO-oxidation	0.326	0.327	0.325	0.327	0.327
Stampino et al.[6]	CNF	TEMPO-oxidation and homogenisation	0.149	0.150	0.180	0.150	0.150
Stampino et al.[6]	CNF	TEMPO-oxidation and ultrasonication	0.206	0.208	0.208	0.207	0.208
Li et al.[7]	CNF	TEMPO-oxidation and homogenisation	0.180	0.184	0.178	0.184	0.185
Li et al.[7]	CNF	TEMPO-oxidation and sonication	1.196	1.201	1.194	1.200	1.201
Arvidsson et al.[5]	CNF	Carboxymethylation	0.015	0.016	0.014	0.016	0.016
Arvidsson et al.[5]	CNF	Enzymatic	0.004	0.005	0.003	0.004	0.005
Stampino et al.[6]	CNF	Enzymatic	0.043	0.044	0.041	0.044	0.044
Li et al.[7]	CNF	Etherification and homogenisation	0.168	0.173	0.166	0.172	0.173
Li et al.[7]	CNF	Etherification and sonification	1.185	1.189	1.182	1.189	1.190
Arvidsson et al.[5]	CNF	Homogenization - low energy	0.021	0.022	0.020	0.021	0.022
Arvidsson et al.[5]	CNF	Homogenization - medium energy	0.027	0.029	0.027	0.028	0.029
Arvidsson et al.[5]	CNF	Homogenization - high energy	0.031	0.032	0.030	0.031	0.032
Sun et al.[8]	CNF	Mechanical - high energy	0.006	0.007	0.005	0.007	0.007
Sun et al.[8]	CNF	Mechanical - low energy	0.002	0.003	0.002	0.003	0.004

Table S31. CO emissions (in kg CO / kg nanocellulose) for the 115 combinations of feedstock and process modeled.

**Process study**	**Cellulose**	**Process**	**Bleached kraft pulp**	**Cotton Liners**	**Industrial Waste**	**Kraft pulp**	**Thermo-mechanical pulp**
Gu et al.[1]	CNC	Acid hydrolysis	0.037	0.047	0.031	0.035	0.033
Teh et al.[2]	CNC	Acid hydrolysis I	0.241	0.246	0.239	0.240	0.240
Teh et al.[2]	CNC	Acid hydrolysis II	0.032	0.037	0.030	0.031	0.031
de Figueiredo et al.[3]	CNC	Acid hydrolysis	0.177	0.186	0.172	0.175	0.174
Zagar et al.[4]	CNC	Acid hydrolysis	0.021	0.037	0.013	0.018	0.016
Zagar et al.[4]	CNC	DES pretreatment - minimum	0.059	0.066	0.055	0.057	0.057
Zagar et al.[4]	CNC	DES pretreatment - average	0.138	0.146	0.134	0.136	0.135
Zagar et al.[4]	CNC	DES pretreatment - maximum	0.253	0.262	0.249	0.251	0.250
Teh et al.[2]	CNC	TEMPO-oxidation	0.797	0.801	0.794	0.795	0.795
Stampino et al.[6]	CNF	TEMPO-oxidation and homogenisation	0.058	0.063	0.071	0.057	0.056
Stampino et al.[6]	CNF	TEMPO-oxidation and ultrasonication	0.078	0.084	0.081	0.077	0.076
Li et al.[7]	CNF	TEMPO-oxidation and homogenisation	1.553	1.572	1.543	1.549	1.547
Li et al.[7]	CNF	TEMPO-oxidation and sonication	1.885	1.904	1.875	1.881	1.878
Arvidsson et al.[5]	CNF	Carboxymethylation	0.050	0.054	0.047	0.049	0.048
Arvidsson et al.[5]	CNF	Enzymatic	0.005	0.009	0.002	0.004	0.003
Stampino et al.[6]	CNF	Enzymatic	0.018	0.023	0.015	0.017	0.016
Li et al.[7]	CNF	Etherification and homogenisation	1.693	1.711	1.683	1.689	1.686
Li et al.[7]	CNF	Etherification and sonification	2.024	2.043	2.014	2.020	2.018
Arvidsson et al.[5]	CNF	Homogenization - low energy	0.009	0.014	0.007	0.008	0.008
Arvidsson et al.[5]	CNF	Homogenization - medium energy	0.011	0.016	0.009	0.010	0.010
Arvidsson et al.[5]	CNF	Homogenization - high energy	0.012	0.017	0.010	0.011	0.011
Sun et al.[8]	CNF	Mechanical - high energy	0.005	0.009	0.002	0.004	0.003
Sun et al.[8]	CNF	Mechanical - low energy	0.003	0.008	0.001	0.002	0.002

Table S32. Pb emissions (in mg Pb / kg nanocellulose) for the 115 combinations of feedstock and process modeled.

Process study	Cellulose	Process	Bleached kraft pulp	Cotton Liners	Industrial Waste	Kraft pulp	Thermo-mechanical pulp
Gu et al.[1]	CNC	Acid hydrolysis	49.95	50.99	47.08	48.23	48.79
Teh et al.[2]	CNC	Acid hydrolysis I	181.92	182.39	180.63	181.15	181.40
Teh et al.[2]	CNC	Acid hydrolysis II	39.64	40.11	38.35	38.87	39.12
de Figueiredo et al.[3]	CNC	Acid hydrolysis	243.20	244.11	240.70	241.70	242.19
Zagar et al.[4]	CNC	Acid hydrolysis	44.32	45.88	40.03	41.74	42.58
Zagar et al.[4]	CNC	DES pretreatment - minimum	41.00	41.69	39.11	39.86	40.23
Zagar et al.[4]	CNC	DES pretreatment - average	71.16	71.96	68.98	69.85	70.28
Zagar et al.[4]	CNC	DES pretreatment - maximum	116.18	117.07	113.75	114.72	115.19
Teh et al.[2]	CNC	TEMPO-oxidation	361.09	361.56	359.81	360.32	360.57
Stampino et al.[6]	CNF	TEMPO-oxidation and homogenisation	72.97	73.47	92.59	72.15	72.41
Stampino et al.[6]	CNF	TEMPO-oxidation and ultrasonication	124.23	126.11	119.08	121.13	122.14
Li et al.[7]	CNF	TEMPO-oxidation and homogenisation	558.28	560.16	553.13	555.18	556.19
Li et al.[7]	CNF	TEMPO-oxidation and sonication	99.93	100.54	107.39	98.93	99.26
Arvidsson et al.[5]	CNF	Carboxymethylation	27.39	27.86	26.10	26.61	26.86
Arvidsson et al.[5]	CNF	Enzymatic	3.12	3.59	1.83	2.34	2.59
Stampino et al.[6]	CNF	Enzymatic	19.72	20.24	17.85	18.86	19.14
Li et al.[7]	CNF	Etherification and homogenisation	134.27	136.15	129.12	131.18	132.18
Li et al.[7]	CNF	Etherification and sonification	568.32	570.20	563.17	565.22	566.23
Arvidsson et al.[5]	CNF	Homogenization - low energy	9.83	10.30	8.54	9.06	9.31
Arvidsson et al.[5]	CNF	Homogenization - medium energy	12.79	13.26	11.50	12.01	12.27
Arvidsson et al.[5]	CNF	Homogenization - high energy	14.09	14.56	12.80	13.32	13.57
Sun et al.[8]	CNF	Mechanical - high energy	3.77	4.24	2.49	3.00	3.25
Sun et al.[8]	CNF	Mechanical - low energy	2.12	2.59	0.83	1.35	1.60

Supplementary References

(1) Gu, H.; Reiner, R.; Bergman, R.; Rudie, A. LCA Study for Pilot Scale Production of Cellulose Nano Crystals (CNC) from Wood Pulp. In *Proceedings from the LCA XV Conference - A bright green future*; Vancouver, British Columbia, Canada, 2015; pp 33–42.

(2) Teh, K. C.; Tan, R. R.; Aviso, K. B.; Promentilla, M. A. B.; Tan, J. An Integrated Analytic Hierarchy Process and Life Cycle Assessment Model for Nanocrystalline Cellulose Production. *Food Bioprod. Process.* **2019**, *118*, 13–31. https://doi.org/https://doi.org/10.1016/j.fbp.2019.08.003.

(3) de Figueirêdo, M. C. B.; Rosa, M. de F.; Ugaya, C. M. L.; Souza Filho, M. de S. M. de; Silva Braid, A. C. C. da; Melo, L. F. L. de. Life Cycle Assessment of Cellulose Nanowhiskers. *J. Clean. Prod.* **2012**, *35*, 130–139. https://doi.org/https://doi.org/10.1016/j.jclepro.2012.05.033.

(4) Zargar, S.; Jiang, J.; Jiang, F.; Tu, Q. Isolation of Lignin-Containing Cellulose Nanocrystals: Life-Cycle Environmental Impacts and Opportunities for Improvement. *Biofuels, Bioprod. Biorefining* **2022**, *16* (1), 68–80. https://doi.org/https://doi.org/10.1002/bbb.2261.

(5) Arvidsson, R.; Nguyen, D.; Svanström, M. Life Cycle Assessment of Cellulose Nanofibrils Production by Mechanical Treatment and Two Different Pretreatment Processes. *Environ. Sci. Technol.* **2015**, *49* (11), 6881–6890. https://doi.org/10.1021/acs.est.5b00888.

(6) Gallo Stampino, P.; Riva, L.; Punta, C.; Elegir, G.; Bussini, D.; Dotelli, G. Comparative Life Cycle Assessment of Cellulose Nanofibres Production Routes from Virgin and Recycled Raw Materials. *Molecules*. 2021. https://doi.org/10.3390/molecules26092558.

(7) Li, Q.; McGinnis, S.; Sydnor, C.; Wong, A.; Renneckar, S. Nanocellulose Life Cycle Assessment. *ACS Sustain. Chem. Eng.* **2013**, *1* (8), 919–928. https://doi.org/10.1021/sc4000225.

(8) Sun, X.-Z ; Moon, D.; Yagishita, T.; Minowa, T.. Evaluation of Energy Consumption and Greenhouse Gas Emissions in Preparation of Cellulose Nanofibers from Woody Biomass. *Trans. ASABE* **2013**, *56* (3), 1061–1067. https://doi.org/https://doi.org/10.13031/trans.56.10040.
